# Histone Methyltransferase SETDB1 Promotes Immune Evasion in Colorectal Cancer *via* FOSB-Mediated Downregulation of MicroRNA-22 through BATF3/PD-L1 Pathway

**DOI:** 10.1155/2022/4012920

**Published:** 2022-04-20

**Authors:** Jiale Tian, Weiwei Wang, Jichao Zhu, Yun Zhuang, Chunrun Qi, Zhengxin Cai, Wenhui Yan, Wenying Lu, Anquan Shang

**Affiliations:** ^1^Department of Laboratory Medicine, Shanghai Tongji Hospital, School of Medicine, Tongji University, Shanghai 200065, China; ^2^Department of Pathology, Tinghu People's Hospital of Yancheng City, Yancheng 224005, China; ^3^Department of Laboratory Medicine, Huzhou Central Hospital, Affiliated Central Hospital of Huzhou Normal University, Huzhou 313003, China; ^4^Department of Immunology, School of Biology and Basic Medical Sciences, Medical College, Soochow University, Suzhou 215123, China; ^5^Department of Laboratory Medicine, The First People's Hospital of Yancheng City, Yancheng 224005, China; ^6^Department of Laboratory Medicine, Tinghu People's Hospital of Yancheng City, Yancheng 224005, China

## Abstract

Tumors may develop a variety of immune evasion mechanisms during the progression of colorectal cancer (CRC). Here, we intended to explore the mechanism of histone methyltransferase SETDB1 in immune evasion in CRC. The expression of SETDB1, microRNA-22 (miR-22), BATF3, PD-L1, and FOSB in CRC tissues and cells was determined with their interactions analyzed also. Gain-of-function and loss-of-function approaches were employed to evaluate the effects of the SETDB1/FOSB/miR-22/BATF3/PD-L1 axis on T cell function, immune cell infiltration, and tumorigenesis. Aberrant high SETDB1 expression in CRC was positively associated with PD-L1 expression. SETDB1 negatively regulated miR-22 expression by downregulating FOSB expression, while miR-22 downregulated PD-L1 expression *via* targeting BATF3. Furthermore, SETDB1 silencing promoted the T cell-mediated cytotoxicity to tumor cells *via* the FOSB/miR-22/BATF3/PD-L1 axis and hindered CRC tumor growth in mice while leading to decreased immune cell infiltration. Taken together, SETDB1 could activate the BATF3/PD-L1 axis by inhibiting FOSB-mediated miR-22 and promote immune evasion in CRC, which provides a better understanding of the mechanisms underlying immune evasion in CRC.

## 1. Introduction

Colorectal cancer (CRC) is the third most common cancer shows higher morbidity in more developed region compared to the less developed region [1]. Emerging evidence has demonstrated that the occurrence and development of CRC could be affected by both genetic and environmental factors, including family history, smoking, drinking, and increased body weight [2]. The currently available treatment approaches include local excision, palliative chemotherapy, downstaging preoperative radiotherapy, systemic therapy, targeted therapy, and immunotherapy based on the CRC patients' conditions [3]. The tumor stage at the time of diagnosis mainly documented that one-third of the CRC patients will die of disease recurrence within five years of diagnosis, while most of them die due to liver metastases [4]. The metastatic process primes cancer cells to escape from the primary tumor, while for cancer resulting from progressive mutations, cancer needs to evade immune recognition and multipronged attacks from tumor antigen-specific T cells [5, 6]. Of note, it is well established that epigenetic aberrations are able to determine tumor development since initiation, including noncoding RNAs, DNA methylation, histone modifications, and chromatin remodeling [7].

SETDB1 (ESET or KMT1E) is an important histone methyltransferase specific for lysine 9 position on histone H3 [8]. SETDB1 (located on human chromosome 1q21.3) shows multiple roles in DNA methylation [9] and several tumors, including lung cancer and CRC [10, 11]. In addition, SETDB1-mediated expression of FOSB/AP-1, a common target gene, is involved in diverse cancers. One study indicated that SETDB1 HMTase was downregulated by several anticancer drugs at the transcriptional level due to its inherent instability. Using RNA sequencing analysis, FOSB was determined to be regulated by SETDB1 during anticancer drug treatment; specifically, FOSB expression increases with the treatment of doxorubicin, paclitaxel, and siSETDB1 [12].

As validated by Starbase database in our current study, we discovered a positive correlation between FOSB and microRNA-22 (miR-22). miRNAs are a noncoding, small RNA molecule known to play significant roles in modulating the metastasis of CRC in each phase, including angiogenesis, invasion, circulation, extravasation, and metastatic colonization [13]. A previous study has documented that miR-22 is downregulated in CRC, and its low expression shares close correlation with liver metastasis [14]. Interestingly, miR-22 is capable of enhancing the sensitivity of CRC cells to 5-FU treatment by limiting cell autophagy and promoting cell apoptosis [15]. More importantly, low expression of FOSB can lead to reduced miR-22, and FOSB can bind to the promoter of miR-22 to promote its transcription [16]. Further, in our study, the bioinformatics prediction indicated that miR-22 could bind to BATF3. BATF3 is a transcription factor that serves as an agent and has been indicated to contribute to the regulation of several molecules such as miR-760 in CRC [17].

In our work, taking account of the correlation and functions of FOSB, miR-22, SETDB1, and BATF3, we speculated that SETDB1 could regulate BATF3 *via* miR-22 and play a role in the immune evasion of CRC.

## 2. Materials and Methods

### 2.1. Ethics Statement

Experimental processes were implemented in the light of the *Declaration of Helsinki* and approved by the Ethics Committee of Shanghai Tongji Hospital. All patients signed informed consent form. All animals in this study were used for medical research purposes, and the procedures were approved by the Animal Ethics Committee of Shanghai Tongji Hospital. Due efforts were achieved to limit animals' pain.

### 2.2. Microarray-Based Gene Expression Profiling

The regulatory pathways of CRC were determined through existing literature. GEPIA was used to analyze colon adenocarcinoma and rectum adenocarcinoma datasets in TCGA database to identify gene expression. Pearson's correlation coefficient was performed to determine the relationship between genes. Gene promoter sequences were obtained from the UCSC database. MethPrimer was applied for prediction of CpG islands in the FOSB promoter sequence, and JASPAR was chose for predicting the binding site between the transcription factor FOSB and miR-22 promoter. Downstream target genes of miR-22 were predicted utilizing miRDB, mirDIP, miRSearch, and TargetScan databases. The prediction results greater than 80 scores from the miRDB database, greater than 0.8 score from the mirDIP database, and the top 100 target genes from the miRSearch and TargetScan databases were subjected to intersection analysis. GENEMANIA database was applied for interaction analysis of candidate target genes. The relationship between transcription factor target genes was retrieved from hTFtarget database.

### 2.3. Tissue Collection

Adjacent normal tissues and CRC tissues were obtained from 36 patients (20 males and 16 females; aged 38-72 years with a mean age of 50 years) who underwent surgery at Shanghai Tongji Hospital from March 2018 to October 2020. Tissue samples were immediately frozen and stored in liquid nitrogen for further use.

### 2.4. Cell Culture

Normal colonic epithelial cell line (FHC, ATCC® CRL-1831), human CRC cells (SW480, ATCC® CCL-228; LS174T, ATCC® CL-188; and HCT114, ATCC® CCL-247), and mouse CRC cells (CT26.WT, ATCC® CRL-2638) were bought from American Type Culture Collection (ATCC) and cultured in Dulbecco's modified Eagle's medium (Thermo Fisher Scientific, Inc., Waltham, MA) appended to 10% fetal bovine serum (FBS, 10100147, Gibco BRL, Invitrogen, CA) at 37°C in a 5% CO_2_-enriched incubator. Neo Jurkat cells (ATCC® CRL-2898) were from the ATCC and cultured in Roswell Park Memorial Institute- (RPMI-) 1640 medium appended to 10% FBS at 37°C in 5% CO_2_.

### 2.5. Lentivirus Infection and Grouping

Cells (logarithmic phase of growth, 4 × 10^5^ cells/well) were seeded into a 6-well culture plate. Under 50-60% confluence, the cells were transduced with lentivirus. A lentiviral packaging system was constructed utilizing overexpression lentiviral-based GV287 vector and GV248 shRNA vector. shRNA and relative shRNA (sh-NC) were constructed by Gene Pharma (Shanghai, China). Cells were transduced with miR-22 mimic, miR-22 inhibitor, sh-SETDB1-1, sh-SETDB1-2, OE-BATF3, OE-SETDB1, OE-FOSB, OE-programmed cell death 1 ligand 1 (PD-L1), and their relative NC together or in combination, respectively. In the light of the viral titer multiplicity of infection (MOI) = 5, appropriate amount of lentivirus was supplemented into the cell culture plate. After mixed, the 6-well plate was placed in a 37°C incubator containing 5% CO_2_ for 24 h before being used for subsequent experiments.

### 2.6. Reverse Transcription-Quantitative Polymerase Chain Reaction (RT-qPCR)

Total RNA from tissues and cells was extracted using TRIzol reagent (15596-018, Solarbio, Beijing, China) and reversely transcribed into cDNA utilizing one-step miRNA reverse transcription kit (D1801, HaiGene, Harbin, China) and cDNA kit (K1622, Beijing Ya An Da Biotechnology Co., Ltd., Beijing, China). RT-qPCR was implemented utilizing fluorescence qPCR instrument (ViiA 7, DAAN Gene Co., Ltd. of Sun Yat-sen University, Guangzhou, China). The transcriptional level of genes was calculated by the relative quantification method (2^-*ΔΔ*Ct^) with glyceraldehyde-phosphate dehydrogenase (GAPDH) and U6 used as internal references [18]. Primers for RT-qPCR are exhibited in Table S1.

### 2.7. Western Blot Analysis

Western blot analysis was implemented as the described previously [19] with the following antibodies purchased from Abcam (Cambridge, UK): rabbit polyclonal SETDB1 (ab228575, 1 : 500), rabbit polyclonal FOSB (ab184938, 1 : 1000), rabbit polyclonal BATF3 (ab98141, 1 : 1000), rabbit polyclonal GAPDH (ab181602, 1 : 5000), rabbit anti-human PD-L1 (ab205921, 1 : 1000), and horseradish peroxidase-labeled goat anti-rabbit immunoglobulin G (IgG; ab205718, 1 : 10000). Protein quantitative analysis was implemented utilizing ImageJ 1.48u software (National Institutes of Health, Maryland).

### 2.8. Immunohistochemistry (IHC)

CRC tissues were prepared into paraffin sections, which were heated in a 60°C incubator for 2 h, dewaxed with xylene, hydrated with gradient alcohol, and reacted in 0.01 mM citrate buffer for 15 to 20 min. After cooling, the sections were blocked with goat serum at ambient temperature for 20 min. IHC was implemented utilizing following antibodies (each for 50 *μ*L, from Abcam, Cambridge, UK): primary antibodies to SETDB1 (ab12317, 1 : 200), BATF3 (ab211304, 1 : 100), Ki-67 (ab15580, 1 : 100), PD-L1 (ab205921, 1 : 100), CD4 (ab213215, 1 : 100), and CD8 (ab4055, 1 : 200), and 50 *μ*L secondary antibody to IgG (ab6728, 1 : 1000). The samples were placed in streptavidin-peroxidase dropwise at 37°C for 30 min and subsequently developed with diaminobenzidine for 5-10 min. The reaction was terminated by washing under running water for 10 min. The samples were then stained with hematoxylin for 2 min, differentiated with hydrochloric acid alcohol, and washed with running water for 10 min, followed by dehydration, clearing, and sealing. Observation was performed under a microscope with the pictures scored by professional pathologists.

### 2.9. Chromatin Immunoprecipitation (ChIP)

ChIP assay was conducted to examine the enrichment of SETDB1 in the FOSB promoter region using ChIP kit (17371; Millipore, Billerica, MA, USA) [20] with rabbit antibody to SETDB1 (ab12317, 1 : 500, Abcam) and H3K9me3 (ab8898, 1 : 200, Abcam). The promoter sequence of FOSB in the complex was quantified by qPCR.

### 2.10. Luciferase Assay

The BATF3 3′ untranslated region (UTR) containing binding sites of wild type (WT; PmirGLO-BATF3-WT) and mutant (MUT) dual-luciferase reporter plasmid (PmirGLO-BATF3-MUT) was provided by GenePharma (Shanghai, China). The mimic NC and miR-22 mimic were, respectively, cotransfected with PmirGLO-BATF3-WT and PmirGLO-BATF3-MUT into 293 T cells (Procell Life Science & Technology Co., Ltd., Wuhan, Hubei, China) and cultured for 48 h. OE-NC, OE-SETDB1, sh-NC, and sh-SETDB1 were cotransfected into 293 T cells and cultured for 48 h. Alteration of the luciferase activity was measured following the manufacturer's instructions of the Dual-Luciferase assay kit (Promega, Madison, WI, USA) applying a GLomax20/20 Luminometer (E5311, Shan Xi Zhong Mei Biotech Co., Ltd., Xi'an, China).

### 2.11. Coculture Experiments and Cytokine Measurement

SW480 cells were inoculated into a 12-well plate (2 × 10^4^ cells/well) for incubation in a 37°C incubator overnight to allow cell attachment. The Jurkat cells were transduced with control or PD-1-expressing lentivirus (GeneChem, Shanghai, China). PD-1 expression in cell surface was measured by Fluorescence-activated Cell Sorting (FACS) analysis. The Jurkat cells were activated by adding 100 ng/mL of CD3 antibody (317303, BioLegend, San Diego, CA) and 100 ng/mL of CD28 antibody (302913, BioLegend, San Diego, CA) and subsequently cocultured with SW480 cells for 72 h. The supernatants were collected and centrifugated, and the secreted interleukin-2 (IL-2) in culture medium was tested utilizing an IL-2 ELISA kit (VAL110, R&D Systems, Minnesota).

### 2.12. Cytotoxicity Assay

After CRC cells were cocultured with T cells (Jurkat), the supernatant was gained for detection of the activity of released lactic dehydrogenase (LDH) utilizing the LDH assay kit (BC0685, Solarbio) to evaluate the cytotoxicity of T cells to CRC cells.

### 2.13. CCK-8 Assay

After 48 h of transfection, the cells were digested and resuspended, and the cell concentration was adjusted to 1 × 10^5^ cells/mL. The cells were inoculated into 96-well plates at 100 *μ*L/well and cultured overnight. Cell viability was tested utilizing CCK-8 kit (Beyotime, Shanghai, China). Thereafter, 10 *μ*L CCK-8 solution was added to each test and incubated in the incubator for 4 h. The absorbance at 450 nm was tested utilizing a microplate reader, and the growth curve was drawn.

### 2.14. Tumor-Bearing Mouse Model

Totally 30 Specific Pathogen Free (SPF) BALB/c nude mice (4 weeks, 18-25 g, irrespective of gender) were bought from Experimental Animal Center of Shanghai Tongji Hospital and maintained under SPF conditions. Mice were submitted to subcutaneous injection of 1 × 10^6^ of cells that transduced with sh-NC+OE-NC, sh-SETDB1-1+OE-NC or sh-SETDB1-1+OE-BATF3 (*n* = 10). The tumor volume was checked every 5 days after inoculation and calculated as follows: *V* (mm^3^) = (*A* × *B*^2^)/2 (*V*: volume, *A*: length, and *B*: width). After experiment, mice were euthanized *via* injection of 3% pentobarbital sodium.

### 2.15. Flow Cytometry

The cells were dispersed into a single-cell suspension and resuspended in staining buffer (BD, New Jersey, USA) while T cells (Jurkat) were treated with PE-CD3 (130-117-139, 1 : 50, Miltenyi Biotec, Bergisch Gladbach, Germany), fixed, permeabilized, and stained with Pacific blue-IFN-*γ* (BioLegend, San Diego, CA, #505817, rat, 1 : 50), followed by detection utilizing flow cytometer BD FACS Canto II (Immunocytometry Systems, BD, New Jersey). Acquired data were analyzed by the Flow Jo software [21].

### 2.16. Statistical Analysis

All data were processed with the help of the SPSS 21.0 statistical software (IBM Corp. Armonk, NY, USA). Measurement data were demonstrated as mean ± standard deviation. Data between CRC tissues and adjacent normal tissues were analyzed by paired *t*-test while the data comparison between the two groups obeying normal distribution and homogeneity of variance was implemented utilizing unpaired *t*-test. Comparison among multiple groups was implemented applying one-way analysis of variance with Tukey's post hoc test. The correlation between SETDB1 and PD-L1 was analyzed by the Pearson's correlation coefficient. ^∗^*p* < 0.05 was concluded as statistically significant. All experiments were devised with three replicates.

## 3. Results

### 3.1. High SETDB1 Expression in CRC Is Positively Associated with PD-L1 Expression

**Figure 1:**
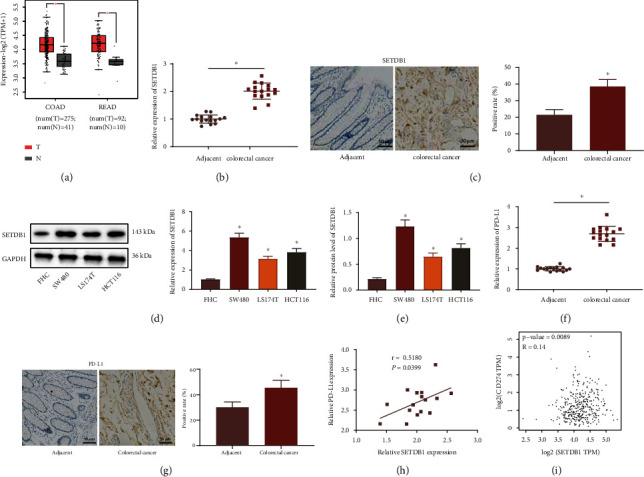
Upregulation of SETDB1 in CRC is positively associated with PD-L1 expression. (a) The expression of SETDB1 in adjacent normal and CRC tissues in TCGA database analyzed by GEPIA. (b) mRNA expression of SETDB1 in CRC tissues and adjacent normal tissues determined by RT-qPCR (*n* = 36). (c) Protein expression of SETDB1 in CRC tissues and adjacent normal tissues determined by IHC (*n* = 36), scale bar: 50 *μ*m. (d) mRNA expression of SETDB1 in FHC and CRC cell lines determined by RT-qPCR. (e) Protein expression of SETDB1 in FHC and CRC cell lines determined by RT-qPCR. (f) mRNA expression of PD-L1 in CRC tissues and adjacent normal tissues determined by RT-qPCR (*n* = 36). (g) Protein expression of PD-L1 in CRC tissues and adjacent normal tissues determined by IHC (*n* = 36), scale bar: 50 *μ*m. (h) Correlation between PD-L1 expression and SETDB1 expression in CRC tissues analyzed by Pearson's correlation coefficient. (i) The correlation between the expression of SETDB1 and PD-L1 (CD274) in CRC based on TCGA database. In (b, c, f, g), ^∗^*p* < 0.05*vs.* adjacent normal tissues. In (d, e), ^∗^*p* < 0.05*vs.* FHC cell line. The cell experiment was run in triplicate independently.

Elevated SETDB1 has been observed in CRC [22], and its upregulation is able to induce proliferation of CRC cells and tumor growth in CRC [10]. Therefore, we speculated that SETDB1 might be involved in immune evasion of CRC. We first found an increase in SETDB1 in CRC samples in TCGA database (Figure 1(a)), which was verified utilizing RT-qPCR and IHC in clinical CRC tissues (Figures 1(b) and 1(c)). Moreover, higher SETDB1 mRNA and protein expression was seen in three human CRC cell lines (SW480, LS174T, and HCT116) relative to FHC cell line, while the highest expression was observed in the SW480 cell line and the lowest expression in the FHC cell line (Figures 1(d) and 1(e)). We therefore used SW480 cells to construct SETDB1 knockdown models and FHC cells to construct SETDB1 overexpression models.

As proteins crucial to cancer immune evasion, PD-L1 has been found upregulation in various cancer [23, 24]. We also identified higher PD-L1 in CRC tissues (Figures 1(f) and 1(g)). In order to investigate whether PD-L1 was associated with SETDB1, the correlation between PD-L1 and SETDB1 in the CRC tissues was analyzed by Pearson's correlation coefficient. Results demonstrated positive correlation between the expression of PD-L1 and SETDB1 (Figure 1(h)). In addition, we analyzed the correlation between the expression of PD-L1 and SETDB1 in CRC based on TCGA data. Spearman analysis found that there was a significant positive correlation between the expression of PD-L1 and SETDB1 (Figure 1(i)). Taken together, these results indicated that SETDB1 was highly expressed in CRC tissues and cells and showed a positive relation with PD-L1 expression, suggesting that SETDB1 may be related to the promotion of CRC immune evasion.

### 3.2. SETDB1 Inhibits miR-22 Expression via Promotion of FOSB Methylation and Downregulation of FOSB Expression

**Figure 2:**
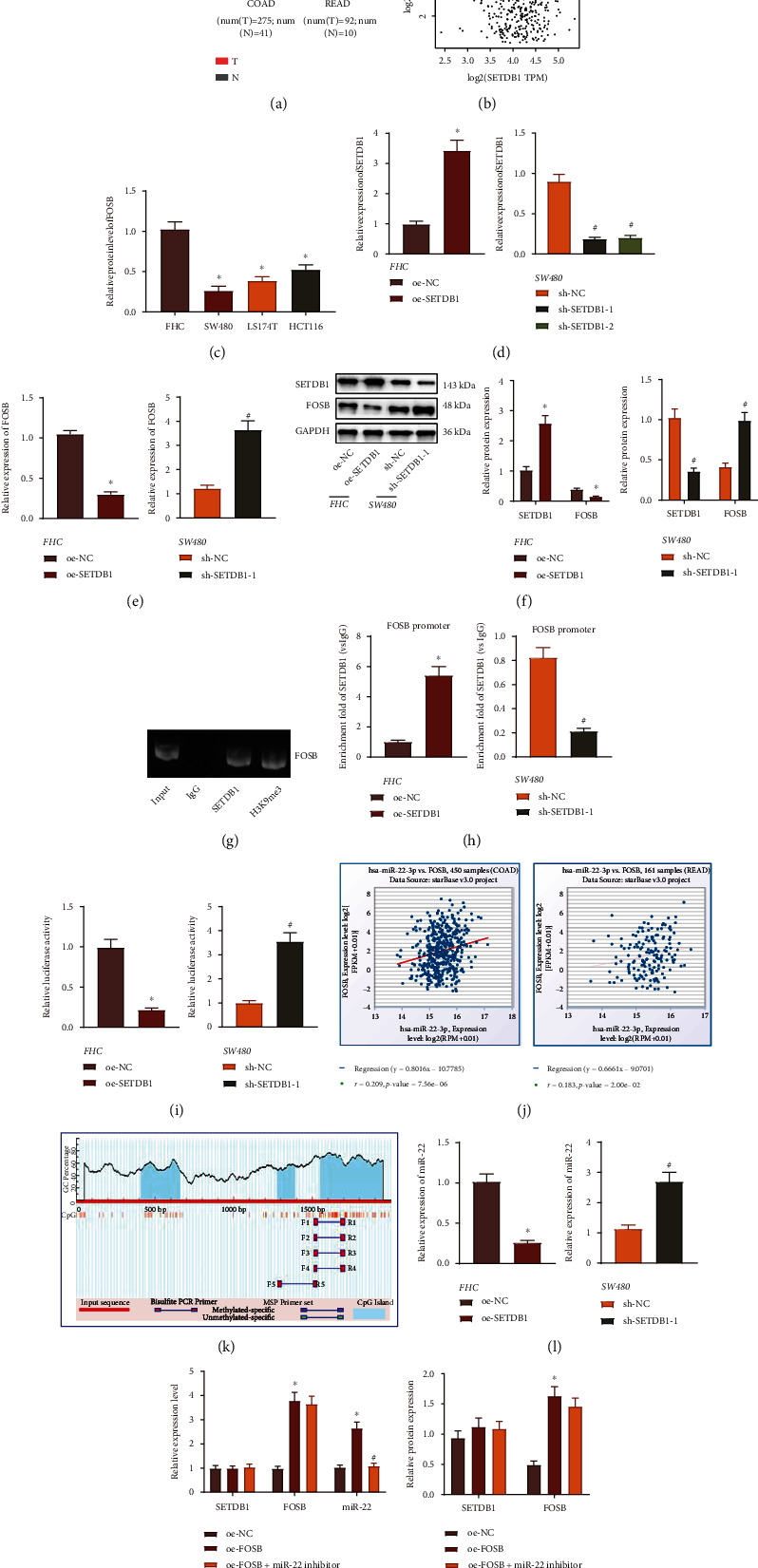
SETDB1 reduces miR-22 expression by downregulating FOSB. (a) The expression of FOSB in adjacent normal and CRC tissues in TCGA database analyzed by GEPIA. (b) The correlation between SETDB1 and FOSB expression was analyzed by GEPIA. (c) FOSB expression in FHC and CRC cell lines determined by RT-qPCR. (d) SETDB1 expression determined by RT-qPCR in SW480 cells treated with sh-SETDB1-1 or sh-SETDB1-2. (e) FOSB expression determined by RT-qPCR in OE-SETDB1-treated FHC cells and sh-SETDB1-treated SW480 cells. (f) Protein expression of FOSB and SETDB1 normalized to GAPDH determined by Western blot analysis in oe-SETDB1-treated FHC cells and sh-SETDB1-treated SW480 cells. (g) The enrichment of SETDB1 and H3K9me3 in the FOSB promoter region in cells detected by ChIP assay. (h) The enrichment of SETDB1 in FOSB promoter region in OE-SETDB1-treated FHC cells and sh-SETDB1-treated SW480 cells detected by ChIP assay. (i) The effect of SETDB1 on the activity of the FOSB promoter region detected by dual-luciferase reporter assay. (j) Starbase was used to analyze the correlation between FOSB and miR-22 in COAD and READ datasets. (k) MethPrimer was used to analyze CpG island methylation in FOSB promoter. (l) The expression of miR-22 determined by RT-qPCR in OE-SETDB1-treated FHC cells and sh-SETDB1-treated SW480 cells. (m) The expression of SETDB1, FOSB, and miR-22 determined by RT-qPCR in cells treated with OE-FOSB or combined with miR-22 inhibitor. (n) Protein expression of FOSB and SETDB1 normalized to GAPDH in cells treated with OE-FOSB or combined with miR-22 inhibitor determined by Western blot assay. ^∗^*p* < 0.05*vs.* FHC cell line or FHC cells treated with OE-NC or SW480 cells treated with sh-NC. ^#^*p* < 0.05*vs.* SW480 cells treated with OE-FOSB or sh-NC. The cell experiment was run in triplicate independently.

Literature has shown that SETDB1 regulates FOSB methylation and then its transcription [12], while the expression of FOSB is low in CRC [25]. Through GEPIA, we found a decline in FOSB expression in CRC samples in TCGA database (Figure 2(a)), and SETDB1 shared negative correlation with FOSB in CRC tissues (Figure 2(b)). The expression of FOSB was decreased in three human CRC cell lines compared to FHC cell line, with the SW480 cell line exhibiting the lowest FOSB expression (Figure 2(c)).

The expression of SETDB1 was silenced in the SW480 cell line, and the silencing efficiency of sh-SETDB1-1 and sh-SETDB1-2 was determined by RT-qPCR. sh-SETDB1-1 with the better efficiency was chosen for the following experiments (Figure 2(d)). Additionally, a reduction of FOSB expression was demonstrated in cells treated with OE-SETDB1 while significant elevation of FOSB was observed in cells treated with sh-SETDB1 (Figures 2(e) and 2(f)). ChIP assay results showed increased enrichment of SETDB1 and H3K9me3 in the FOSB promoter (Figure 2(g)). Meanwhile, FOSB promoter enriched by SETDB1 was enhanced in the presence of OE-SETDB1 while decreased upon sh-SETDB1 (Figure 2(h)). Moreover, dual-luciferase reporter assay results revealed that the luciferase activity was limited following overexpression of STEDB1 while silencing of SETDB1 caused an enhancement (Figure 2(i)). Taken together, SETDB1 was suggested to bind to the FOSB promoter region, thus inhibiting FOSB transcription.

In chronic myeloid leukemia, FOSB could bind to the promoter of miR-22 and promotes its transcription, and knockdown of FOSB could inhibit the expression of miR-22 [16]. As validated by Starbase, FOSB was positively correlated with miR-22 expression (Figure 2(j)). The promoter sequence of FOSB was obtained from UCSC, and CpG island was found on FOSB promoter by MethPrimer analysis (Figure 2(k)). The promoter sequence of miR-22 was obtained by UCSC, and the transcription binding sites of FOSB and miR-22 promoter were gained by JASPAR analysis (Table S2). Lower miR-22 has been discovered in CRC [26]. Thus, we speculated that SETDB1 could limit the expression of miR-22 by promoting FOSB methylation, thereby affecting CRC. RT-qPCR results showed that OE-SETDB1 treatment limited miR-22 expression while sh-SETDB1 treatment elevated its expression (Figure 2(l)). Besides, SW480 cells treated with OE-FOSB exhibited increased expression of FOSB and miR-22, whereas SETDB1 expression remained unchanged in comparison with SW480 cells treated with OE-NC. SW480 cells treated with OE-FOSB+miR-22 inhibitor showed reduced expression of miR-22 while no difference was seen regarding the expression of SETDB1 and FOSB in comparison with SW480 cells treated with OE-FOSB (Figure 2(m)). Western blot analysis yielded similar results to RT-qPCR (Figure 2(n)). Taken together, the abovementioned results pinpointed that SETDB1 could promote FOSB methylation, inhibit the expression of FOSB, and then reduce the expression of miR-22.

### 3.3. miR-22 Downregulates PD-L1 Expression via Targeting BATF3 in CRC Cells

**Figure 3:**
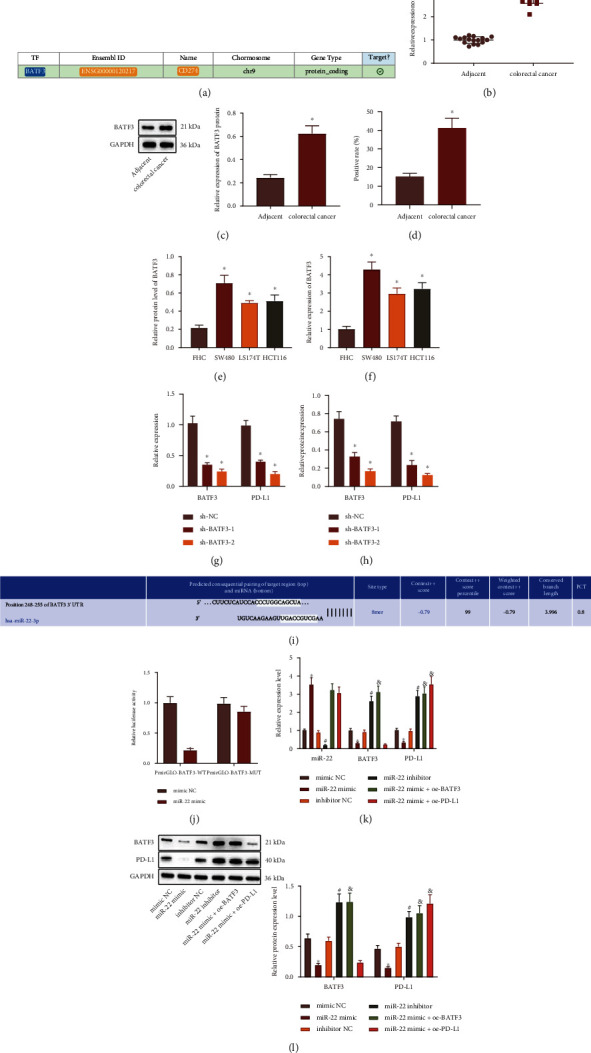
miR-22 diminishes PD-L1 expression *via* targeting BATF3 in CRC cells. (a) The binding relationship between BATF3 and PD-L1 (namely, CD274) analyzed by hTFtarget database. (b) mRNA expression of BATF3 in CRC tissues and adjacent normal tissues determined by RT-qPCR. (c) Protein expression of BATF3 in CRC tissues and adjacent normal tissues determined by Western blot analysis. (d) The expression of BATF3 in CRC tissues and adjacent normal tissues determined by IHC. (e) Protein expression of BATF3 in FHC and CRC cell lines determined by Western blot analysis. (f) mRNA expression of BATF3 in FHC and CRC cell lines determined by RT-qPCR. (g) mRNA expression of PD-L1 in SW480 cells treated with sh-BATF3-1 or sh-BATF3-2 determined by RT-qPCR. (h) Protein expression of PD-L1 in SW480 cells treated with sh-BATF3-1 or sh-BATF3-2 determined by Western blot analysis. (i) The binding site of miR-22 in the 3′UTR of BATF3 mRNA predicted using TargetScan website. (j) The binding of miR-22 to BATF3 confirmed by dual-luciferase reporter assay. (k) The expression of miR-22, BATF3, and PD-L1 in SW480 cells treated with miR-22 mimic, miR-22 inhibitor, miR-22 mimic+OE-BATF3, or miR-22 mimic+OE-PD-L1 determined by RT-qPCR. (l) The protein expression of BATF3 and PD-L1 normalized to GAPDH in SW480 cells treated with miR-22 mimic, miR-22 inhibitor, miR-22 mimic+OE-BATF3, or miR-22 mimic+OE-PD-L1 determined by Western blot analysis. ^∗^*p* < 0.05*vs.* adjacent normal tissues, FHC cell line, HEK293T cells transfected with mimic NC, or SW480 cells treated with sh-NC or mimic NC. ^#^*p* < 0.05*vs.* SW480 cells treated with inhibitor NC. ^&^*p* < 0.05*vs.* SW480 cells treated with miR-22 mimic. The cell experiment was run in triplicate independently.

Based on hTFtarget database, BATF3 exerted regulatory action in PD-L1 (also known as CD274) (Figure 3(a)). BATF3 has been pinpointed to elevate PD-L1 expression [27]. To identify the role of BATF3 in regulating PD-L1 expression, we designed two sequences to silence BATF3 in SW480 cells. It was evident that BATF3 expression was higher in CRC tissues than that in adjacent normal tissues (Figures 3(b)–3(d)). Furthermore, higher BATF3 expression was also confirmed in CRC cell lines than that in FHC cell line (Figures 3(e) and 3(f)). In SW480 cells treated with sh-BATF3-1 or sh-BATF3-2, we found reduced PD-L1 mRNA and protein expression (Figures 3(g) and 3(h)).

The TargetScan database predicted the presence of binding sites between miR-22 and BATF3 (Figure 3(i)). Luciferase assay revealed that cotransfection of miR-22 mimic and PmirGLO-BATF3-WT led to a reduction in luciferase activity in comparison with cells treated with mimic NC, while no significant difference was observed after the cotransfection of miR-22 mimic and PmirGLO-BATF3-MUT (Figure 3(j)). Additionally, upon miR-22 mimic transfection, miR-22 expression was increased, but mRNA and protein expression of BATF3 and PD-L1 was downregulated, while miR-22 inhibitor caused contrary findings. Relative to miR-22 mimic transfection alone, miR-22 mimic+OE-BATF3 transfection enhanced BATF3 and PD-L1 expression, while miR-22 mimic+OE-PD-L1 transfection elevated PD-L1 expression (Figures 3(k) and 3(l)). Based on the abovementioned results, miR-22 could target BATF3 and negatively regulate BATF3 expression, thus limiting PD-L1 in CRC cells.

### 3.4. SETDB1 Silencing Inhibits PD-L1 Expression and Promotes the T Cell-Mediated Cytotoxicity to Tumor Cells via the FOSB/miR-22/BATF3 Axis

**Figure 4:**
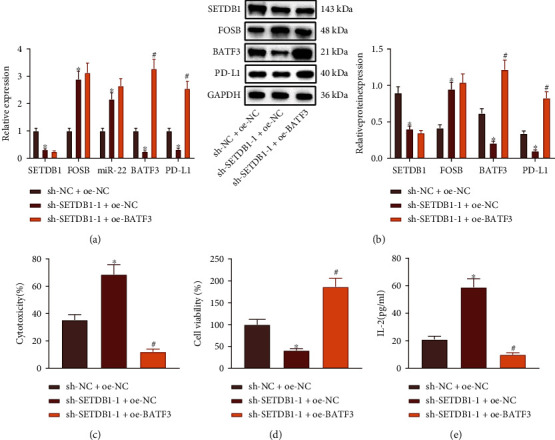
Silencing of SETDB1 inhibits PD-L1 expression and facilitates the T cell-mediated cytotoxicity to tumor cells *via* the FOSB/miR-22/BATF3 axis. (a) The expression of SETDB1, FOSB, miR-22, BATF3, and PD-L1 in SW480 cells treated with sh-SETDB1 or combined with OE-BATF3 determined by RT-qPCR. (b) The protein expression of SETDB1, FOSB, BATF3, and PD-L1 in SW480 cells treated with sh-SETDB1 or combined with OE-BATF3 normalized to GAPDH determined by Western blot analysis. (c) T cell cytotoxicity determined by the LDH test kit following treatment with sh-SETDB1 or combined with OE-BATF3. (d) The viability of SW480 cells treated with sh-SETDB1 or combined with OE-BATF3 detected by CCK-8 assay. (e) The content of IL-2 in the cell supernatant following treatment with sh-SETDB1 or combined with OE-BATF3 determined by ELISA. ^∗^*p* < 0.05*vs.* SW480 cells treated with sh-NC+OE-NC. ^#^*p* < 0.05*vs.* SW480 cells treated with sh-SETDB1-1+OE-NC. The cell experiment was run in triplicate independently.

Next, we aimed to pinpoint the regulatory role of SETDB1 in CRC. We found that the expression of SETDB1, BATF3, and PD-L1 was diminished while that of FOSB and miR-22 was upregulated upon sh-SETDB1 treatment. Expression of BATF3 and PD-L1 was increased in cells treated with sh-SETDB1+OE-BATF3 relative to sh-SETDB1+OE-NC treatment (Figures 4(a) and 4(b)).

Additionally, the SW480 cell cytotoxicity was increased, and the SW480 cell viability was decreased following treatment with sh-SETDB1, while further OE-BATF3 treatment displayed opposite results (Figures 4(c) and 4(d)). Moreover, elevated IL-2 was found in SW480 cells treated with sh-SETDB1 but was decreased after further OE-BATF3 treatment (Figure 4(e)). In conclusion, silencing of SETDB1 could inhibit PD-L1 expression in CRC cells *via* the FOSB/miR-22/BATF3 axis and exerted the PD-L1/PD-1-mediated immunosuppression, leading to enhanced cytotoxicity of T cells.

### 3.5. SETDB1 Silencing Hinders CRC Tumor Growth and Immune Cell Infiltration in Mice

**Figure 5:**
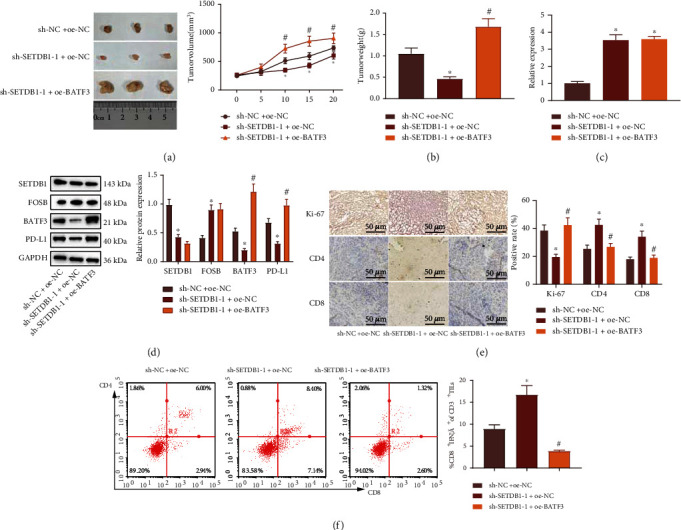
Downregulation of SETDB1 represses CRC tumor growth and immune cell infiltration in mice *via* the FOSB/miR-22/BATF3/PD-L1 axis. (a) Tumor volume in nude mice after different treatments. (b) Tumor weight in nude mice after different treatments. (c) miR-22 expression in tumor tissues of nude mice determined by RT-qPCR. (d) The protein expression of SETDB1, FOSB, BATF3, and PD-L1 in tumor tissues of nude mice normalized to GAPDH determined by Western blot analysis. (e) The expression of Ki-67, CD4, and CD8 in tumor tissues of nude mice determined by IHC, scale bar: 50 *μ*m. (f) Percentage of CD8^+^ and IFN-*γ*^+^ in tumor tissues of nude mice analyzed by flow cytometry. ^∗^*p* < 0.05*vs.* nude mice bearing cells treated with sh-NC+OE-NC. ^#^*p* < 0.05*vs.* nude mice bearing cells treated with sh-SETDB1+OE-NC. *n* = 10.

Finally, we sought to understand the effect of SETDB1 on the pathogenesis of CRC *via* the FOSB/miR-22/BATF3 axis *in vivo*. Tumor weight and volume of mice injected with SW480 cells harboring sh-SETDB1-1+OE-NC were reduced, while increased after sh-SETDB1-1+OE-BATF3 treatment (Figures 5(a) and 5(b)).

We also noted elevated miR-22 in the tumor tissues of mice treated with sh-SETDB1-1+OE-NC while no significant difference in the miR-22 expression after further addition of OE-BATF3 (Figure 5(c)). Additionally, the protein expression of SETDB1, BATF3, and PD-L1 was diminished while FOSB expression was upregulated in the presence of sh-SETDB1+OE-NC while opposite results were detected in the tumor tissues of mice treated with sh-SETDB1+OE-BATF3 (Figure 5(d)). The abovementioned results suggested that SETDB1 could regulate PD-L1 *via* the FOSB/miR-22/BATF3 axis *in vivo*.

IHC results noted that the expression of Ki-67 was downregulated while that of CD4 and CD8 was upregulated upon sh-SETDB1, while further OE-BATF3 brought about opposite trends (Figure 5(e)). In addition, flow cytometric analysis results demonstrated that the percentage of IFN-*γ*^+^ and CD8^+^ was elevated in response to sh-SETDB1, while further OE-BATF3 led to contrary findings (Figure 5(f)). The results indicated that silencing of SETDB1 limited tumor cell proliferation and the infiltration of tumor cells by T cells *via* FOSB/miR-22/BATF3/PD-L1 axis and consequently arresting tumor cell immune evasion.

## 4. Discussion

CRC is a common cancer, mainly characterized by bloody stools, bowel habit change, and abdominal pain with metastatic potential [2]. However, with increasing incidence, current treatments hardly decline the mortality and improve patients' prognosis [2]. Complete comprehension of the mechanism of CRC progression becomes more urgent for developing optimal treatment. Emerging evidence has revealed the multifunctional role of histone methyltransferase SETDB1 in diseases such as breast cancer and cellular processes [9, 28]. Here, we focused on investigating the regulatory role of SETDB1 in CRC. Consequently, our work demonstrated that SETDB1 could activate the BATF3/PD-L1 axis through the inhibition of miR-22 expression and thus promote immune evasion in CRC.

Initially, the present study unfolded that SETDB1 was upregulated in CRC tissues and cell lines. Consistent with our study, previous finding also confirmed elevated SETDB1 in CRC [29]. PD-L1 has been reported to play regulatory role in immune response to cancer and tends to be the subject of immunotherapy [30]. In addition, PD-L1 expression is a negative prognostic factor in cancer [31]. Consistent with previous findings, we also reported that PD-L1 was highly expressed in CRC tissues. The study done by Ho et al. also indicated higher expression of PD-L1 in CRC [22]. Apart from these indexes, the transcription factor BATF3 was also found upregulated in our study. Another study also showed the high BATF3 expression in CRC [17]. More importantly, BATF3 has been suggested to reduce T cell activity and induce immune reaction [32].

It is worthy to note that STEDB1 could inhibit miR-22 expression through FOSB expression downregulation in our study. FOSB/AP-1 has been proved as an ideal target of gene therapy for CRC with great biological properties [33]. It has also been reported that FOSB could exert a functional role in modulating the proliferation of cancer cells through regulating miR-22 expression, suggesting the potential targeting relationship between FOSB and miR-22 [16]. Interestingly, the relationship between SETDB1 and FOSB has also been elaborated in another report, whereby the silencing of SETDB1 could upregulate FOSB expression [12].

Additionally, the interaction among PD-L1, BATF3 and miR-22 was clarified in the present study. The essential role of BATF3 in the up-regulation of PD-L1 has been demonstrated recently [27]. Previous evidence unveiled that miR-22 was downregulated in CRC and the up-regulation of miR-22 could hinder proliferation, metastasis, and epithelial-mesenchymal transition (EMT) [26] Meanwhile, EMT score reflecting the possibility of metastasis was positively correlated with PD-L1 expression in tumors [34]. Based on the above-mentioned evidence, we speculate that miR-22 could result in the down-regulation of PD-L1 expression through decreasing BATF3 in CRC.

Moreover, we demonstrated that silencing of STEDB1 contributed to reduced PD-L1 expression in CRC cells, exerting immunosuppressive activity mediated by checkpoint PD-L1/PD-1, and resulted in enhanced T cell function. As earlier described, the upregulation of PD-L1 in the tumor microenvironment *in vivo* hinged on CD8^+^ T cells and PD-L1 participated in immune evasion by inhibiting immune cells [35]. Anti-PD-L1 antibody has been demonstrated to lead to potent tumor-specific T cell immune responses, as well as inhibition of tumor growth [36]. The restoration of BATF3 recruitment and activation of tumor microenvironment could contribute to effector T cell accumulation and clinical activity [37]. Furthermore, miR-22 has been shown to be upregulated in emphysema and antigen-presenting cells, suggesting that miR-22 could control the T cell responses through the activation of transcription factor complexes [38]. Besides, miR-22 is able to expand the stem cell compartment and exerts crucial action in tumor development [39]. Thus, the present study further demonstrated that silencing of STEDB1 could dramatically downregulate the expression of PD-L1, thereby promoting T cell cytotoxicity to tumor cells *via* the FOSB/miR-22/BATF3 axis.

Finally, *in vivo* experimental results revealed that SETDB1 silencing could impede CRC tumor growth in mice and immune cell infiltration *via* the FOSB/miR-22/BATF3/PD-L1 axis. SETDB1 has been reported to suppress proinflammatory cytokine expression and disrupt normal immune reaction in the previous study [40]. Besides, SETDB1 has been shown to allow cell escape from the immune-mediated control and proliferate in cancers of breast, liver, and prostate [28, 41, 42]. In addition, a study done by Chen et al. also demonstrated that a significant increase in malignant properties of CRC cells was observed *in vitro* and tumor growth *in vivo* in case of forced expression of SETDB1 [10].

## 5. Conclusion

**Figure 6:**
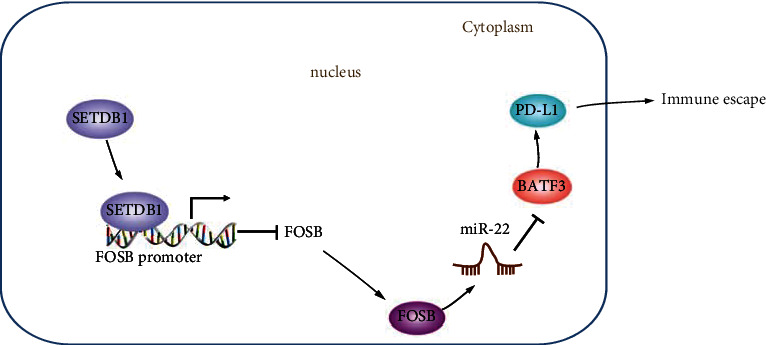
The schematic diagram depicts the molecular mechanism by which SETDB1 stimulates the immune evasion in CRC *via* the FOSB/miR-22/BATF3/PD-L1 axis. SETDB1 promotes FOSB methylation and inhibits FOSB expression, thereby reducing the expression of miR-22, enhancing BATF3 protein expression, and increasing PD-L1 expression in CRC cells. By this mechanism, SETDB1 augments the proliferation of CRC cells and inhibits their apoptosis as well as attenuating the killing effect of T cells on tumors, thereby favoring the immune escape of CRC.

Taken altogether, our study demonstrated that SETDB1 could inhibit miR-22 expression which could contribute to elevated PD-L1. SETDB1 could also inhibit T cell cytotoxicity and promote tumor growth and immune cell infiltration *via* the FOSB/miR-22/BATF3/PD-L1 axis (Figure 6). This study provides a promising SETDB1-targeted therapeutic strategy and provided a better understanding of the molecular mechanism underlying SETDB1 in promoting immune evasion in CRC. More in-depth investigation on the potential role of SETDB1 in CRC is critically needed as the molecular mechanism remains relatively unexplored poorly characterized.

## Data Availability

The data that support the findings of this study are available from the corresponding author upon reasonable request.

## Supplementary Materials

Supplementary Materials Table S1: primer sequences. Table S2: transcription binding site of FOSB and miR-22 promoter.
