# Supplementary material for: Histone Methyltransferase SETDB1 Promotes Immune Evasion in Colorectal Cancer via FOSB-Mediated Downregulation of MicroRNA-22 through BATF3/PD-L1 Pathway
Source: J Immunol Res. 2022 Apr 20;2022:4012920. doi: 10.1155/2022/4012920 (PMC9045983; doi:10.1155/2022/4012920)
Supplement: Supplementary Materials — Table S1: primer sequences. Table S2: transcription binding site of FOSB and miR-22 promoter. [file 4012920.f1.docx]

**Table S1** Primer sequences

| Gene | Primer |
| --- | --- |
| SETDB1 | F: 5´-AGGAACTTCGGCATTTCATCG-3´ |
|  | R: 5´-TGTCCCGGTATTGTAGTCCCA-3´ |
| FOSB | F: 5´-GACCCCGAGAGGAGACGCTCAC-3´ |
|  | R: 5´-CAACTGATCTGTCTCCGCCTGG-3´ |
| miR-22 | F: 5´-AGTTCTTCAGTGGCAAGCTTTA-3´ |
|  | R: Universal primer |
| BATF3 | F: 5´-ACAGGAAGGTCCGAAGGAGA-3´ |
|  | R: 5´-CCAGGCTCTCATATTCCTCATGG-3´ |
| PD-L1 | F: 5´-TGCCGACTACAAGCGAATTACTG-3´ |
|  | R: 5´-CTGCTTGTCCAGATGACTTCGG-3´ |
| U6 | F: 5´-GTGCTCGCTTCGGCAGCACATA-3′ |
|  | R: Universal primer |
| GAPDH | F: 5´-ACAACTTTGGTATCGTGGAAGG-3′ |
|  | R: 5´-GCCATCACGCCACAGTTTC-3′ |

Note: F: forward; R: reverse.

**Table S2** Transcription binding site of FOSB and miR-22 promoter

| Matrix ID | Name | Score | Relative score | Sequence ID | Start | End | Strand | Predicted sequence |
| --- | --- | --- | --- | --- | --- | --- | --- | --- |
| UN0122.1 | FOSB | 2.07093 | 0.740275703 | MIR22 | 663 | 674 | - | GGTGACAGCCTC |
| UN0122.1 | FOSB | 2.0676 | 0.740227154 | MIR22 | 663 | 674 | + | GAGGCTGTCACC |
| UN0122.1 | FOSB | 1.23736 | 0.728136755 | MIR22 | 315 | 326 | - | CATAACATCCCC |
| UN0122.1 | FOSB | 1.03442 | 0.725181386 | MIR22 | 315 | 326 | + | GGGGATGTTATG |
| UN0122.1 | FOSB | 0.45544 | 0.716750024 | MIR22 | 320 | 331 | - | CCTTACATAACA |
| UN0122.1 | FOSB | -0.064169 | 0.709183201 | MIR22 | 320 | 331 | + | TGTTATGTAAGG |
| UN0122.1 | FOSB | -0.674063 | 0.700301601 | MIR22 | 1115 | 1126 | + | GCTCACATCCCC |
| UN0122.1 | FOSB | -0.781753 | 0.698733361 | MIR22 | 485 | 496 | + | GAGAAGGTCACC |
| UN0122.1 | FOSB | -0.810225 | 0.698318742 | MIR22 | 1115 | 1126 | - | GGGGATGTGAGC |
| UN0122.1 | FOSB | -0.869441 | 0.697456402 | MIR22 | 485 | 496 | - | GGTGACCTTCTC |
| UN0122.1 | FOSB | -1.98029 | 0.681279653 | MIR22 | 1088 | 1099 | - | CGTGCTGACACT |
| UN0122.1 | FOSB | -2.07289 | 0.679931135 | MIR22 | 1988 | 1999 | + | CACGCCCTCACC |
| UN0122.1 | FOSB | -2.1833 | 0.678323261 | MIR22 | 969 | 980 | - | AATTCCGAAACC |
| UN0122.1 | FOSB | -2.34082 | 0.676029398 | MIR22 | 1988 | 1999 | - | GGTGAGGGCGTG |
| UN0122.1 | FOSB | -2.45843 | 0.674316633 | MIR22 | 1592 | 1603 | + | ACTGAACTCACA |
| UN0122.1 | FOSB | -2.60697 | 0.672153545 | MIR22 | 1985 | 1996 | - | GAGGGCGTGAGA |
| UN0122.1 | FOSB | -2.7158 | 0.670568789 | MIR22 | 1088 | 1099 | + | AGTGTCAGCACG |
| UN0122.1 | FOSB | -3.04235 | 0.665813352 | MIR22 | 1985 | 1996 | + | TCTCACGCCCTC |
| UN0122.1 | FOSB | -3.28714 | 0.662248598 | MIR22 | 969 | 980 | + | GGTTTCGGAATT |
| UN0122.1 | FOSB | -3.66961 | 0.656678785 | MIR22 | 718 | 729 | - | CAGGATGACAGG |
| UN0122.1 | FOSB | -3.91944 | 0.653040714 | MIR22 | 59 | 70 | - | CCTTCCGCCACG |
| UN0122.1 | FOSB | -4.03301 | 0.651386774 | MIR22 | 59 | 70 | + | CGTGGCGGAAGG |
| UN0122.1 | FOSB | -4.08147 | 0.650681171 | MIR22 | 747 | 758 | - | TCTGACAGCTCC |
| UN0122.1 | FOSB | -4.18766 | 0.649134745 | MIR22 | 369 | 380 | - | GGTTGCATAAGG |
| UN0122.1 | FOSB | -4.19565 | 0.649018413 | MIR22 | 1592 | 1603 | - | TGTGAGTTCAGT |
| UN0122.1 | FOSB | -4.20053 | 0.648947314 | MIR22 | 1588 | 1599 | - | AGTTCAGTGATC |
| UN0122.1 | FOSB | -4.23896 | 0.648387707 | MIR22 | 715 | 726 | - | GATGACAGGCCA |
| UN0122.1 | FOSB | -4.34961 | 0.646776286 | MIR22 | 718 | 729 | + | CCTGTCATCCTG |
| UN0122.1 | FOSB | -4.36831 | 0.646503957 | MIR22 | 369 | 380 | + | CCTTATGCAACC |
| UN0122.1 | FOSB | -4.40119 | 0.646025151 | MIR22 | 747 | 758 | + | GGAGCTGTCAGA |
| UN0122.1 | FOSB | -4.53131 | 0.644130284 | MIR22 | 1411 | 1422 | + | CCTGCCGATACC |
| UN0122.1 | FOSB | -4.61318 | 0.642938062 | MIR22 | 1570 | 1581 | + | GAACAAGTCAGT |
| UN0122.1 | FOSB | -4.67193 | 0.642082497 | MIR22 | 715 | 726 | + | TGGCCTGTCATC |
| UN0122.1 | FOSB | -4.67913 | 0.641977734 | MIR22 | 590 | 601 | + | GGGTTAGTCATC |
| UN0122.1 | FOSB | -4.79726 | 0.640257445 | MIR22 | 490 | 501 | - | GCAGAGGTGACC |
| UN0122.1 | FOSB | -4.83146 | 0.639759369 | MIR22 | 1729 | 1740 | - | CCTGCTCTCACC |
| UN0122.1 | FOSB | -4.89199 | 0.638877924 | MIR22 | 590 | 601 | - | GATGACTAACCC |
| UN0122.1 | FOSB | -4.97616 | 0.63765219 | MIR22 | 524 | 535 | - | AGGGGCCTCACA |
| UN0122.1 | FOSB | -5.03068 | 0.636858176 | MIR22 | 490 | 501 | + | GGTCACCTCTGC |
| UN0122.1 | FOSB | -5.08471 | 0.636071405 | MIR22 | 1729 | 1740 | + | GGTGAGAGCAGG |
| UN0122.1 | FOSB | -5.18853 | 0.634559546 | MIR22 | 1570 | 1581 | - | ACTGACTTGTTC |
| UN0122.1 | FOSB | -5.21679 | 0.634148026 | MIR22 | 225 | 236 | - | AACGCCGGCAGA |
| UN0122.1 | FOSB | -5.23505 | 0.633882108 | MIR22 | 1479 | 1490 | + | GCTGACCTTCTT |
| UN0122.1 | FOSB | -5.2461 | 0.633721091 | MIR22 | 225 | 236 | + | TCTGCCGGCGTT |
| UN0122.1 | FOSB | -5.27571 | 0.633289955 | MIR22 | 359 | 370 | + | GGTGCCGGGGCC |
| UN0122.1 | FOSB | -5.34993 | 0.632209142 | MIR22 | 1250 | 1261 | - | TCTGCCGGCCCC |
| UN0122.1 | FOSB | -5.36083 | 0.632050396 | MIR22 | 1479 | 1490 | - | AAGAAGGTCAGC |
| UN0122.1 | FOSB | -5.38294 | 0.631728495 | MIR22 | 1250 | 1261 | + | GGGGCCGGCAGA |
| UN0122.1 | FOSB | -5.41912 | 0.631201567 | MIR22 | 359 | 370 | - | GGCCCCGGCACC |
| UN0122.1 | FOSB | -5.58613 | 0.628769425 | MIR22 | 1588 | 1599 | + | GATCACTGAACT |
| UN0122.1 | FOSB | -5.61864 | 0.628296097 | MIR22 | 1411 | 1422 | - | GGTATCGGCAGG |
| UN0122.1 | FOSB | -5.62017 | 0.628273703 | MIR22 | 47 | 58 | - | ACTCCCGTCCCA |
| UN0122.1 | FOSB | -5.65855 | 0.627714874 | MIR22 | 524 | 535 | + | TGTGAGGCCCCT |
| UN0122.1 | FOSB | -5.74398 | 0.626470739 | MIR22 | 1612 | 1623 | + | CCTGAGGTACCA |
| UN0122.1 | FOSB | -5.77753 | 0.625982197 | MIR22 | 1612 | 1623 | - | TGGTACCTCAGG |
| UN0122.1 | FOSB | -5.80283 | 0.625613772 | MIR22 | 47 | 58 | + | TGGGACGGGAGT |
| UN0122.1 | FOSB | -5.98602 | 0.62294607 | MIR22 | 388 | 399 | - | GGTGAAACCCTA |
| UN0122.1 | FOSB | -6.03712 | 0.622201914 | MIR22 | 1061 | 1072 | + | CCTGGCGTGGGC |
| UN0122.1 | FOSB | -6.18625 | 0.620030212 | MIR22 | 550 | 561 | - | CAGGCAGTCAGG |
| UN0122.1 | FOSB | -6.32694 | 0.61798137 | MIR22 | 1133 | 1144 | - | GGGGGCGTGGCA |
| UN0122.1 | FOSB | -6.34675 | 0.617692974 | MIR22 | 1061 | 1072 | - | GCCCACGCCAGG |
| UN0122.1 | FOSB | -6.54173 | 0.614853437 | MIR22 | 1929 | 1940 | + | CATGCCCTGCTC |
| UN0122.1 | FOSB | -6.56004 | 0.614586851 | MIR22 | 1373 | 1384 | - | GGGGACCACAGG |
| UN0122.1 | FOSB | -6.56004 | 0.614586851 | MIR22 | 1437 | 1448 | - | GGGGACCACAGG |
| UN0122.1 | FOSB | -6.71794 | 0.612287513 | MIR22 | 1236 | 1247 | + | AGTTCAGACATG |
| UN0122.1 | FOSB | -6.74465 | 0.611898408 | MIR22 | 209 | 220 | - | AAGCACCTCCTC |
| UN0122.1 | FOSB | -6.7717 | 0.611504519 | MIR22 | 209 | 220 | + | GAGGAGGTGCTT |
| UN0122.1 | FOSB | -6.81609 | 0.610858072 | MIR22 | 1929 | 1940 | - | GAGCAGGGCATG |
| UN0122.1 | FOSB | -6.83765 | 0.610544225 | MIR22 | 71 | 82 | + | GCTGGCGGAGTG |
| UN0122.1 | FOSB | -6.89019 | 0.609779113 | MIR22 | 650 | 661 | + | CATGGCCTCCGG |
| UN0122.1 | FOSB | -6.91981 | 0.609347726 | MIR22 | 1133 | 1144 | + | TGCCACGCCCCC |
| UN0122.1 | FOSB | -6.97873 | 0.608489606 | MIR22 | 650 | 661 | - | CCGGAGGCCATG |
| UN0122.1 | FOSB | -7.04303 | 0.607553345 | MIR22 | 388 | 399 | + | TAGGGTTTCACC |
| UN0122.1 | FOSB | -7.17387 | 0.605647923 | MIR22 | 17 | 28 | - | CCTGACACACTA |
| UN0122.1 | FOSB | -7.19089 | 0.605400045 | MIR22 | 593 | 604 | - | AGTGATGACTAA |
| UN0122.1 | FOSB | -7.19568 | 0.605330328 | MIR22 | 550 | 561 | + | CCTGACTGCCTG |
| UN0122.1 | FOSB | -7.19781 | 0.605299358 | MIR22 | 71 | 82 | - | CACTCCGCCAGC |
| UN0122.1 | FOSB | -7.28337 | 0.604053376 | MIR22 | 1556 | 1567 | + | GCTGAACTCCCT |
| UN0122.1 | FOSB | -7.30008 | 0.603809997 | MIR22 | 17 | 28 | + | TAGTGTGTCAGG |
| UN0122.1 | FOSB | -7.37267 | 0.602752863 | MIR22 | 1814 | 1825 | - | GTTAACCTCGCT |
| UN0122.1 | FOSB | -7.37744 | 0.602683458 | MIR22 | 54 | 65 | - | CGCCACGACTCC |
| UN0122.1 | FOSB | -7.5748 | 0.599809458 | MIR22 | 1814 | 1825 | + | AGCGAGGTTAAC |
| UN0122.1 | FOSB | -7.60459 | 0.59937551 | MIR22 | 1373 | 1384 | + | CCTGTGGTCCCC |
| UN0122.1 | FOSB | -7.60459 | 0.59937551 | MIR22 | 1437 | 1448 | + | CCTGTGGTCCCC |
| UN0122.1 | FOSB | -7.64815 | 0.598741221 | MIR22 | 1907 | 1918 | - | GCTGACCCACTC |
| UN0122.1 | FOSB | -7.69971 | 0.597990426 | MIR22 | 1278 | 1289 | - | GGGTACATCTGG |
| UN0122.1 | FOSB | -7.70115 | 0.597969393 | MIR22 | 1907 | 1918 | + | GAGTGGGTCAGC |
| UN0122.1 | FOSB | -7.73449 | 0.597483969 | MIR22 | 147 | 158 | + | TCTGAGGATACA |
| UN0122.1 | FOSB | -7.7461 | 0.597314786 | MIR22 | 694 | 705 | + | GGTTCCAGCCTC |
| UN0122.1 | FOSB | -7.77264 | 0.596928307 | MIR22 | 917 | 928 | - | GGTGACCAGTCC |
| UN0122.1 | FOSB | -7.78061 | 0.596812259 | MIR22 | 426 | 437 | - | CAGGCCCTCCTC |
| UN0122.1 | FOSB | -7.79345 | 0.596625349 | MIR22 | 666 | 677 | - | GGGGGTGACAGC |
| UN0122.1 | FOSB | -7.81866 | 0.596258187 | MIR22 | 694 | 705 | - | GAGGCTGGAACC |
| UN0122.1 | FOSB | -7.84508 | 0.59587345 | MIR22 | 1278 | 1289 | + | CCAGATGTACCC |
| UN0122.1 | FOSB | -7.94004 | 0.59449063 | MIR22 | 467 | 478 | + | CTGGCCGTCTTG |
| UN0122.1 | FOSB | -7.94262 | 0.594452966 | MIR22 | 426 | 437 | + | GAGGAGGGCCTG |
| UN0122.1 | FOSB | -8.02711 | 0.593222581 | MIR22 | 415 | 426 | - | CTTGCCCTCTTC |
| UN0122.1 | FOSB | -8.0727 | 0.592558766 | MIR22 | 467 | 478 | - | CAAGACGGCCAG |
| UN0122.1 | FOSB | -8.0967 | 0.592209235 | MIR22 | 769 | 780 | - | TGCTCCATCAAC |
| UN0122.1 | FOSB | -8.12249 | 0.591833581 | MIR22 | 1309 | 1320 | + | CCTGGCATCTGA |
| UN0122.1 | FOSB | -8.17903 | 0.591010208 | MIR22 | 391 | 402 | - | CACGGTGAAACC |
| UN0122.1 | FOSB | -8.19639 | 0.590757434 | MIR22 | 415 | 426 | + | GAAGAGGGCAAG |
| UN0122.1 | FOSB | -8.21266 | 0.590520479 | MIR22 | 54 | 65 | + | GGAGTCGTGGCG |
| UN0122.1 | FOSB | -8.23916 | 0.590134617 | MIR22 | 593 | 604 | + | TTAGTCATCACT |
| UN0122.1 | FOSB | -8.25988 | 0.589832819 | MIR22 | 1780 | 1791 | + | AGGGAAGTCTGT |
| UN0122.1 | FOSB | -8.26303 | 0.58978703 | MIR22 | 20 | 31 | - | GCTCCTGACACA |
| UN0122.1 | FOSB | -8.26585 | 0.589745908 | MIR22 | 769 | 780 | + | GTTGATGGAGCA |
| UN0122.1 | FOSB | -8.31152 | 0.589080844 | MIR22 | 299 | 310 | - | GAGAACGAGAGG |
| UN0122.1 | FOSB | -8.32915 | 0.588824153 | MIR22 | 1918 | 1929 | + | CGTTGCAGCAAC |
| UN0122.1 | FOSB | -8.37936 | 0.588092955 | MIR22 | 1393 | 1404 | + | CCTCACAGCCCC |
| UN0122.1 | FOSB | -8.37936 | 0.588092955 | MIR22 | 1457 | 1468 | + | CCTCACAGCCCC |
| UN0122.1 | FOSB | -8.43083 | 0.587343466 | MIR22 | 1393 | 1404 | - | GGGGCTGTGAGG |
| UN0122.1 | FOSB | -8.43083 | 0.587343466 | MIR22 | 1457 | 1468 | - | GGGGCTGTGAGG |
| UN0122.1 | FOSB | -8.53458 | 0.58583253 | MIR22 | 286 | 297 | - | GGGGCCGGCCCG |
| UN0122.1 | FOSB | -8.58742 | 0.585063084 | MIR22 | 286 | 297 | + | CGGGCCGGCCCC |
| UN0122.1 | FOSB | -8.64906 | 0.584165508 | MIR22 | 666 | 677 | + | GCTGTCACCCCC |
| UN0122.1 | FOSB | -8.65062 | 0.58414269 | MIR22 | 1309 | 1320 | - | TCAGATGCCAGG |
| UN0122.1 | FOSB | -8.6598 | 0.584009075 | MIR22 | 147 | 158 | - | TGTATCCTCAGA |
| UN0122.1 | FOSB | -8.6752 | 0.583784716 | MIR22 | 1918 | 1929 | - | GTTGCTGCAACG |
| UN0122.1 | FOSB | -8.6903 | 0.58356487 | MIR22 | 1556 | 1567 | - | AGGGAGTTCAGC |
| UN0122.1 | FOSB | -8.79374 | 0.582058476 | MIR22 | 935 | 946 | + | AAGGAAGATACA |
| UN0122.1 | FOSB | -8.81193 | 0.581793703 | MIR22 | 1820 | 1831 | + | GTTAACAGCTTC |
| UN0122.1 | FOSB | -8.82826 | 0.581555858 | MIR22 | 1616 | 1627 | + | AGGTACCACACT |
| UN0122.1 | FOSB | -8.83531 | 0.581453185 | MIR22 | 905 | 916 | - | AACTCCGTCCGA |
| UN0122.1 | FOSB | -8.84031 | 0.581380287 | MIR22 | 905 | 916 | + | TCGGACGGAGTT |
| UN0122.1 | FOSB | -8.87517 | 0.580872753 | MIR22 | 951 | 962 | - | GGAGGCATGAAC |
| UN0122.1 | FOSB | -8.92909 | 0.580087475 | MIR22 | 1236 | 1247 | - | CATGTCTGAACT |
| UN0122.1 | FOSB | -8.94589 | 0.57984284 | MIR22 | 120 | 131 | + | GGGGGCGGCTCA |
| UN0122.1 | FOSB | -9.0025 | 0.579018495 | MIR22 | 271 | 282 | - | AGCGACCCCTTC |
| UN0122.1 | FOSB | -9.08827 | 0.577769374 | MIR22 | 271 | 282 | + | GAAGGGGTCGCT |
| UN0122.1 | FOSB | -9.09321 | 0.577697504 | MIR22 | 1820 | 1831 | - | GAAGCTGTTAAC |
| UN0122.1 | FOSB | -9.10506 | 0.577524961 | MIR22 | 1257 | 1268 | + | GCAGACAGCCTG |
| UN0122.1 | FOSB | -9.19299 | 0.576244481 | MIR22 | 917 | 928 | + | GGACTGGTCACC |
| UN0122.1 | FOSB | -9.22023 | 0.575847717 | MIR22 | 391 | 402 | + | GGTTTCACCGTG |
| UN0122.1 | FOSB | -9.24689 | 0.57545955 | MIR22 | 1780 | 1791 | - | ACAGACTTCCCT |
| UN0122.1 | FOSB | -9.29571 | 0.574748586 | MIR22 | 20 | 31 | + | TGTGTCAGGAGC |
| UN0122.1 | FOSB | -9.37177 | 0.573641025 | MIR22 | 1257 | 1268 | - | CAGGCTGTCTGC |
| UN0122.1 | FOSB | -9.37453 | 0.573600778 | MIR22 | 727 | 738 | - | TCTGACAGCCAG |
| UN0122.1 | FOSB | -9.39739 | 0.573267898 | MIR22 | 1894 | 1905 | - | CTGGCCATCAGG |
| UN0122.1 | FOSB | -9.40979 | 0.573087314 | MIR22 | 1266 | 1277 | + | CTGGAATTCATA |
| UN0122.1 | FOSB | -9.41637 | 0.572991529 | MIR22 | 120 | 131 | - | TGAGCCGCCCCC |
| UN0122.1 | FOSB | -9.42147 | 0.572917215 | MIR22 | 1796 | 1807 | + | AGCCACCTCTTG |
| UN0122.1 | FOSB | -9.42156 | 0.572915895 | MIR22 | 951 | 962 | + | GTTCATGCCTCC |
| UN0122.1 | FOSB | -9.42938 | 0.572802001 | MIR22 | 1894 | 1905 | + | CCTGATGGCCAG |
| UN0122.1 | FOSB | -9.51369 | 0.571574309 | MIR22 | 1070 | 1081 | - | AAGGGCATGGCC |
| UN0122.1 | FOSB | -9.54175 | 0.571165629 | MIR22 | 1266 | 1277 | - | TATGAATTCCAG |
| UN0122.1 | FOSB | -9.55611 | 0.570956464 | MIR22 | 299 | 310 | + | CCTCTCGTTCTC |
| UN0122.1 | FOSB | -9.56082 | 0.570887843 | MIR22 | 364 | 375 | + | CGGGGCCTTATG |
| UN0122.1 | FOSB | -9.5653 | 0.570822723 | MIR22 | 931 | 942 | + | GGGGAAGGAAGA |
| UN0122.1 | FOSB | -9.57326 | 0.5707068 | MIR22 | 1796 | 1807 | - | CAAGAGGTGGCT |
| UN0122.1 | FOSB | -9.59896 | 0.570332535 | MIR22 | 883 | 894 | + | AGCTATCTCAGA |
| UN0122.1 | FOSB | -9.60285 | 0.570275914 | MIR22 | 727 | 738 | + | CTGGCTGTCAGA |
| UN0122.1 | FOSB | -9.63302 | 0.569836528 | MIR22 | 883 | 894 | - | TCTGAGATAGCT |
| UN0122.1 | FOSB | -9.64114 | 0.569718245 | MIR22 | 1506 | 1517 | - | GAGGAAGGGAGG |
| UN0122.1 | FOSB | -9.66121 | 0.569425946 | MIR22 | 1676 | 1687 | - | CATTCAGACAGG |
| UN0122.1 | FOSB | -9.68577 | 0.569068332 | MIR22 | 1937 | 1948 | + | GCTCAGATCTTT |
| UN0122.1 | FOSB | -9.69196 | 0.56897813 | MIR22 | 1937 | 1948 | - | AAAGATCTGAGC |
| UN0122.1 | FOSB | -9.7312 | 0.568406795 | MIR22 | 1093 | 1104 | - | GGGTCCGTGCTG |
| UN0122.1 | FOSB | -9.81285 | 0.567217726 | MIR22 | 1416 | 1427 | - | AGGGAGGTATCG |
| UN0122.1 | FOSB | -9.84167 | 0.56679795 | MIR22 | 1416 | 1427 | + | CGATACCTCCCT |
| UN0122.1 | FOSB | -9.87926 | 0.566250613 | MIR22 | 364 | 375 | - | CATAAGGCCCCG |
| UN0122.1 | FOSB | -9.94726 | 0.565260405 | MIR22 | 237 | 248 | - | CCTGACCCAGTG |
| UN0122.1 | FOSB | -9.99984 | 0.564494626 | MIR22 | 248 | 259 | - | GCTGAACTGGCC |
| UN0122.1 | FOSB | -10.0002 | 0.56448964 | MIR22 | 988 | 999 | - | GGATACGGGGTG |
| UN0122.1 | FOSB | -10.0131 | 0.564301736 | MIR22 | 995 | 1006 | - | GATTGGGGGATA |
| UN0122.1 | FOSB | -10.0157 | 0.564263795 | MIR22 | 1093 | 1104 | + | CAGCACGGACCC |
| UN0122.1 | FOSB | -10.0205 | 0.564193674 | MIR22 | 1476 | 1487 | + | TCTGCTGACCTT |
| UN0122.1 | FOSB | -10.0313 | 0.564036255 | MIR22 | 237 | 248 | + | CACTGGGTCAGG |
| UN0122.1 | FOSB | -10.1056 | 0.56295422 | MIR22 | 988 | 999 | + | CACCCCGTATCC |
| UN0122.1 | FOSB | -10.1207 | 0.562733944 | MIR22 | 1616 | 1627 | - | AGTGTGGTACCT |
| UN0122.1 | FOSB | -10.133 | 0.56255497 | MIR22 | 1039 | 1050 | - | AAGGCGGTCTTG |
| UN0122.1 | FOSB | -10.1899 | 0.561727542 | MIR22 | 1128 | 1139 | - | CGTGGCAGGCTC |
| UN0122.1 | FOSB | -10.1972 | 0.561620508 | MIR22 | 1291 | 1302 | - | GCTTGCGCATTC |
| UN0122.1 | FOSB | -10.2311 | 0.56112714 | MIR22 | 1039 | 1050 | + | CAAGACCGCCTT |
| UN0122.1 | FOSB | -10.2352 | 0.5610667 | MIR22 | 1070 | 1081 | + | GGCCATGCCCTT |
| UN0122.1 | FOSB | -10.2461 | 0.560908794 | MIR22 | 1597 | 1608 | + | ACTCACATTTCT |
| UN0122.1 | FOSB | -10.3247 | 0.559764402 | MIR22 | 32 | 43 | + | TACGAGGAAAGA |
| UN0122.1 | FOSB | -10.3255 | 0.559752195 | MIR22 | 1602 | 1613 | - | GGTCCAGAAATG |
| UN0122.1 | FOSB | -10.3602 | 0.55924623 | MIR22 | 823 | 834 | + | GCCAAGGGCACC |
| UN0122.1 | FOSB | -10.3949 | 0.558740905 | MIR22 | 1597 | 1608 | - | AGAAATGTGAGT |
| UN0122.1 | FOSB | -10.3974 | 0.558705018 | MIR22 | 995 | 1006 | + | TATCCCCCAATC |
| UN0122.1 | FOSB | -10.4222 | 0.558343335 | MIR22 | 1924 | 1935 | + | AGCAACATGCCC |
| UN0122.1 | FOSB | -10.47 | 0.557647301 | MIR22 | 1291 | 1302 | + | GAATGCGCAAGC |
| UN0122.1 | FOSB | -10.492 | 0.557327712 | MIR22 | 132 | 143 | + | CCCGGCGGCCCC |
| UN0122.1 | FOSB | -10.5545 | 0.556417929 | MIR22 | 1708 | 1719 | - | CCTAGCGGCCCC |
| UN0122.1 | FOSB | -10.5653 | 0.556260273 | MIR22 | 1128 | 1139 | + | GAGCCTGCCACG |
| UN0122.1 | FOSB | -10.5671 | 0.556234303 | MIR22 | 931 | 942 | - | TCTTCCTTCCCC |
| UN0122.1 | FOSB | -10.605 | 0.555682272 | MIR22 | 823 | 834 | - | GGTGCCCTTGGC |
| UN0122.1 | FOSB | -10.6508 | 0.555014708 | MIR22 | 1034 | 1045 | + | CAAGGCAAGACC |
| UN0122.1 | FOSB | -10.6919 | 0.554417208 | MIR22 | 1849 | 1860 | + | GCTGCAGCCCTG |
| UN0122.1 | FOSB | -10.7379 | 0.553746575 | MIR22 | 350 | 361 | - | ACCGCCGCCCCC |
| UN0122.1 | FOSB | -10.7752 | 0.553202863 | MIR22 | 772 | 783 | + | GATGGAGCAGCT |
| UN0122.1 | FOSB | -10.7984 | 0.552865373 | MIR22 | 1476 | 1487 | - | AAGGTCAGCAGA |
| UN0122.1 | FOSB | -10.833 | 0.552361866 | MIR22 | 350 | 361 | + | GGGGGCGGCGGT |
| UN0122.1 | FOSB | -10.8402 | 0.552256499 | MIR22 | 1417 | 1428 | - | GAGGGAGGTATC |
| UN0122.1 | FOSB | -10.8456 | 0.55217856 | MIR22 | 1506 | 1517 | + | CCTCCCTTCCTC |
| UN0122.1 | FOSB | -10.8509 | 0.552100732 | MIR22 | 859 | 870 | + | TGCTGAGTCCTC |
| UN0122.1 | FOSB | -10.8587 | 0.55198749 | MIR22 | 132 | 143 | - | GGGGCCGCCGGG |
| UN0122.1 | FOSB | -10.8839 | 0.551620127 | MIR22 | 1661 | 1672 | - | GGGCCCGGGACC |
| UN0122.1 | FOSB | -10.9254 | 0.551015961 | MIR22 | 32 | 43 | - | TCTTTCCTCGTA |
| UN0122.1 | FOSB | -10.9529 | 0.550616003 | MIR22 | 1924 | 1935 | - | GGGCATGTTGCT |
| UN0122.1 | FOSB | -10.9663 | 0.550421058 | MIR22 | 79 | 90 | + | AGTGGCGGGGGG |
| UN0122.1 | FOSB | -10.9775 | 0.550258042 | MIR22 | 1661 | 1672 | + | GGTCCCGGGCCC |
| UN0122.1 | FOSB | -10.9896 | 0.550081013 | MIR22 | 1195 | 1206 | - | AGTTCCAACGCA |
| UN0122.1 | FOSB | -10.9924 | 0.55004046 | MIR22 | 1708 | 1719 | + | GGGGCCGCTAGG |
| UN0122.1 | FOSB | -11.0108 | 0.549773229 | MIR22 | 1136 | 1147 | - | GCTGGGGGCGTG |
| UN0122.1 | FOSB | -11.0199 | 0.549640141 | MIR22 | 1919 | 1930 | + | GTTGCAGCAACA |
| UN0122.1 | FOSB | -11.0347 | 0.549424434 | MIR22 | 1687 | 1698 | + | GCTGAGGAGGCT |
| UN0122.1 | FOSB | -11.0664 | 0.548962397 | MIR22 | 935 | 946 | - | TGTATCTTCCTT |
| UN0122.1 | FOSB | -11.0741 | 0.548850724 | MIR22 | 1926 | 1937 | - | CAGGGCATGTTG |
| UN0122.1 | FOSB | -11.1054 | 0.548394798 | MIR22 | 1864 | 1875 | + | TAGAATCTCAGA |
| UN0122.1 | FOSB | -11.1235 | 0.548131302 | MIR22 | 1864 | 1875 | - | TCTGAGATTCTA |
| UN0122.1 | FOSB | -11.1283 | 0.548061293 | MIR22 | 1713 | 1724 | - | CCTGCCCTAGCG |
| UN0122.1 | FOSB | -11.1362 | 0.547945801 | MIR22 | 79 | 90 | - | CCCCCCGCCACT |
| UN0122.1 | FOSB | -11.2253 | 0.546649059 | MIR22 | 1136 | 1147 | + | CACGCCCCCAGC |
| UN0122.1 | FOSB | -11.2308 | 0.546568954 | MIR22 | 847 | 858 | - | GCTGCTGAGAGT |
| UN0122.1 | FOSB | -11.2555 | 0.54620859 | MIR22 | 1099 | 1110 | - | GAGGGCGGGTCC |
| UN0122.1 | FOSB | -11.2704 | 0.545991828 | MIR22 | 796 | 807 | + | AGTGCCCTGGGC |
| UN0122.1 | FOSB | -11.2882 | 0.54573286 | MIR22 | 385 | 396 | - | GAAACCCTAACC |
| UN0122.1 | FOSB | -11.3031 | 0.545515347 | MIR22 | 1883 | 1894 | + | TGGGGCAGGACC |
| UN0122.1 | FOSB | -11.3469 | 0.544878739 | MIR22 | 51 | 62 | + | ACGGGAGTCGTG |
| UN0122.1 | FOSB | -11.4033 | 0.544056811 | MIR22 | 353 | 364 | + | GGCGGCGGTGCC |
| UN0122.1 | FOSB | -11.4152 | 0.543883364 | MIR22 | 796 | 807 | - | GCCCAGGGCACT |
| UN0122.1 | FOSB | -11.4253 | 0.543735653 | MIR22 | 613 | 624 | - | GGTGGGGAGAGA |
| UN0122.1 | FOSB | -11.4282 | 0.543694294 | MIR22 | 1509 | 1520 | - | AGTGAGGAAGGG |
| UN0122.1 | FOSB | -11.4568 | 0.54327774 | MIR22 | 353 | 364 | - | GGCACCGCCGCC |
| UN0122.1 | FOSB | -11.4977 | 0.542681796 | MIR22 | 750 | 761 | - | CACTCTGACAGC |
| UN0122.1 | FOSB | -11.5014 | 0.542627939 | MIR22 | 1607 | 1618 | + | CTGGACCTGAGG |
| UN0122.1 | FOSB | -11.5067 | 0.542550569 | MIR22 | 1713 | 1724 | + | CGCTAGGGCAGG |
| UN0122.1 | FOSB | -11.5497 | 0.541925113 | MIR22 | 1980 | 1991 | - | CGTGAGAGGAAC |
| UN0122.1 | FOSB | -11.5523 | 0.541887185 | MIR22 | 660 | 671 | - | GACAGCCTCGCC |
| UN0122.1 | FOSB | -11.5553 | 0.541842786 | MIR22 | 560 | 571 | + | TGGGAAGCCGGC |
| UN0122.1 | FOSB | -11.5585 | 0.541797136 | MIR22 | 1726 | 1737 | + | GAGGGTGAGAGC |
| UN0122.1 | FOSB | -11.5663 | 0.541683158 | MIR22 | 1921 | 1932 | + | TGCAGCAACATG |
| UN0122.1 | FOSB | -11.5855 | 0.541403289 | MIR22 | 817 | 828 | + | CCTGCAGCCAAG |
| UN0122.1 | FOSB | -11.59 | 0.541338529 | MIR22 | 385 | 396 | + | GGTTAGGGTTTC |
| UN0122.1 | FOSB | -11.5931 | 0.541293157 | MIR22 | 1676 | 1687 | + | CCTGTCTGAATG |
| UN0122.1 | FOSB | -11.6137 | 0.540992067 | MIR22 | 1068 | 1079 | + | TGGGCCATGCCC |
| UN0122.1 | FOSB | -11.6272 | 0.540796512 | MIR22 | 521 | 532 | + | GATTGTGAGGCC |
| UN0122.1 | FOSB | -11.636 | 0.54066734 | MIR22 | 477 | 488 | + | TGGGCCGAGAGA |
| UN0122.1 | FOSB | -11.6622 | 0.540286478 | MIR22 | 1980 | 1991 | + | GTTCCTCTCACG |
| UN0122.1 | FOSB | -11.6937 | 0.539828413 | MIR22 | 248 | 259 | + | GGCCAGTTCAGC |
| UN0122.1 | FOSB | -11.7183 | 0.539470188 | MIR22 | 1607 | 1618 | - | CCTCAGGTCCAG |
| UN0122.1 | FOSB | -11.7415 | 0.539131684 | MIR22 | 1050 | 1061 | - | GCTGAGGGGCCA |
| UN0122.1 | FOSB | -11.7585 | 0.538884771 | MIR22 | 1790 | 1801 | + | GTTTACAGCCAC |
| UN0122.1 | FOSB | -11.7729 | 0.53867423 | MIR22 | 772 | 783 | - | AGCTGCTCCATC |
| UN0122.1 | FOSB | -11.7765 | 0.538621817 | MIR22 | 809 | 820 | - | CAGGGCGGGGTG |
| UN0122.1 | FOSB | -11.7879 | 0.538455593 | MIR22 | 1849 | 1860 | - | CAGGGCTGCAGC |
| UN0122.1 | FOSB | -11.7909 | 0.538412665 | MIR22 | 779 | 790 | - | TCTGACCAGCTG |
| UN0122.1 | FOSB | -11.8036 | 0.538227053 | MIR22 | 563 | 574 | - | CCAGCCGGCTTC |
| UN0122.1 | FOSB | -11.8176 | 0.538023262 | MIR22 | 1926 | 1937 | + | CAACATGCCCTG |
| UN0122.1 | FOSB | -11.8251 | 0.537914922 | MIR22 | 1883 | 1894 | - | GGTCCTGCCCCA |
| UN0122.1 | FOSB | -11.8385 | 0.537719603 | MIR22 | 1099 | 1110 | + | GGACCCGCCCTC |
| UN0122.1 | FOSB | -11.8426 | 0.537659413 | MIR22 | 1790 | 1801 | - | GTGGCTGTAAAC |
| UN0122.1 | FOSB | -11.8595 | 0.537413791 | MIR22 | 1817 | 1828 | - | GCTGTTAACCTC |
| UN0122.1 | FOSB | -11.8694 | 0.537269107 | MIR22 | 332 | 343 | - | TCTCCCCTCCCC |
| UN0122.1 | FOSB | -11.8868 | 0.537015916 | MIR22 | 1068 | 1079 | - | GGGCATGGCCCA |
| UN0122.1 | FOSB | -11.9114 | 0.536657886 | MIR22 | 332 | 343 | + | GGGGAGGGGAGA |
| UN0122.1 | FOSB | -11.9463 | 0.536148699 | MIR22 | 1156 | 1167 | + | TGGGCCATGCTG |
| UN0122.1 | FOSB | -11.9516 | 0.536072371 | MIR22 | 1469 | 1480 | + | GGGGGCCTCTGC |
| UN0122.1 | FOSB | -11.9633 | 0.535901397 | MIR22 | 1509 | 1520 | + | CCCTTCCTCACT |
| UN0122.1 | FOSB | -11.9707 | 0.535793266 | MIR22 | 563 | 574 | + | GAAGCCGGCTGG |
| UN0122.1 | FOSB | -11.9859 | 0.535572656 | MIR22 | 1817 | 1828 | + | GAGGTTAACAGC |
| UN0122.1 | FOSB | -12 | 0.535367629 | MIR22 | 859 | 870 | - | GAGGACTCAGCA |
| UN0122.1 | FOSB | -12.0321 | 0.534900217 | MIR22 | 1244 | 1255 | + | CATGGAGGGGCC |
| UN0122.1 | FOSB | -12.034 | 0.534871553 | MIR22 | 1919 | 1930 | - | TGTTGCTGCAAC |
| UN0122.1 | FOSB | -12.0393 | 0.534795294 | MIR22 | 1050 | 1061 | + | TGGCCCCTCAGC |
| UN0122.1 | FOSB | -12.0495 | 0.534646027 | MIR22 | 1580 | 1591 | + | GTTGGGCTGATC |
| UN0122.1 | FOSB | -12.0711 | 0.534332424 | MIR22 | 560 | 571 | - | GCCGGCTTCCCA |
| UN0122.1 | FOSB | -12.0761 | 0.534259554 | MIR22 | 160 | 171 | - | GATGGCCGGCCC |
| UN0122.1 | FOSB | -12.0913 | 0.534038236 | MIR22 | 1417 | 1428 | + | GATACCTCCCTC |
| UN0122.1 | FOSB | -12.0962 | 0.533966394 | MIR22 | 493 | 504 | - | GGTGCAGAGGTG |
| UN0122.1 | FOSB | -12.1315 | 0.533452652 | MIR22 | 660 | 671 | + | GGCGAGGCTGTC |
| UN0122.1 | FOSB | -12.1457 | 0.533246125 | MIR22 | 1752 | 1763 | - | ACTGGAGTCTAG |
| UN0122.1 | FOSB | -12.163 | 0.532993018 | MIR22 | 750 | 761 | + | GCTGTCAGAGTG |
| UN0122.1 | FOSB | -12.1639 | 0.532979908 | MIR22 | 1647 | 1658 | + | TGGGGTGTGTCC |
| UN0122.1 | FOSB | -12.1643 | 0.5329752 | MIR22 | 1034 | 1045 | - | GGTCTTGCCTTG |
| UN0122.1 | FOSB | -12.1773 | 0.532785144 | MIR22 | 1347 | 1358 | + | CCTGCCCATACC |
| UN0122.1 | FOSB | -12.1872 | 0.532641348 | MIR22 | 1687 | 1698 | - | AGCCTCCTCAGC |
| UN0122.1 | FOSB | -12.2007 | 0.532445251 | MIR22 | 809 | 820 | + | CACCCCGCCCTG |
| UN0122.1 | FOSB | -12.2061 | 0.532365826 | MIR22 | 1602 | 1613 | + | CATTTCTGGACC |
| UN0122.1 | FOSB | -12.2182 | 0.532189269 | MIR22 | 1102 | 1113 | + | CCCGCCCTCCCC |
| UN0122.1 | FOSB | -12.225 | 0.53209047 | MIR22 | 847 | 858 | + | ACTCTCAGCAGC |
| UN0122.1 | FOSB | -12.2288 | 0.532035766 | MIR22 | 1156 | 1167 | - | CAGCATGGCCCA |
| UN0122.1 | FOSB | -12.2366 | 0.531921371 | MIR22 | 1469 | 1480 | - | GCAGAGGCCCCC |
| UN0122.1 | FOSB | -12.2623 | 0.5315473 | MIR22 | 1353 | 1364 | - | GAGGGAGGTATG |
| UN0122.1 | FOSB | -12.2747 | 0.531366466 | MIR22 | 1271 | 1282 | - | TCTGGTATGAAT |
| UN0122.1 | FOSB | -12.2851 | 0.531215754 | MIR22 | 1073 | 1084 | - | TCCAAGGGCATG |
| UN0122.1 | FOSB | -12.2875 | 0.53118052 | MIR22 | 214 | 225 | - | AGGCAAAGCACC |
| UN0122.1 | FOSB | -12.3261 | 0.53061806 | MIR22 | 1837 | 1848 | - | TCTGATCTGGGC |
| UN0122.1 | FOSB | -12.3339 | 0.53050429 | MIR22 | 1195 | 1206 | + | TGCGTTGGAACT |
| UN0122.1 | FOSB | -12.3435 | 0.53036523 | MIR22 | 1837 | 1848 | + | GCCCAGATCAGA |
| UN0122.1 | FOSB | -12.3494 | 0.53027925 | MIR22 | 1414 | 1425 | + | GCCGATACCTCC |
| UN0122.1 | FOSB | -12.3511 | 0.530255016 | MIR22 | 1665 | 1676 | + | CCGGGCCCCACC |
| UN0122.1 | FOSB | -12.3832 | 0.52978741 | MIR22 | 268 | 279 | + | AGGGAAGGGGTC |
| UN0122.1 | FOSB | -12.3931 | 0.529642878 | MIR22 | 1378 | 1389 | + | GGTCCCCTGCCC |
| UN0122.1 | FOSB | -12.4023 | 0.529508152 | MIR22 | 1073 | 1084 | + | CATGCCCTTGGA |
| UN0122.1 | FOSB | -12.4262 | 0.529160051 | MIR22 | 1647 | 1658 | - | GGACACACCCCA |
| UN0122.1 | FOSB | -12.4289 | 0.52912197 | MIR22 | 1580 | 1591 | - | GATCAGCCCAAC |
| UN0122.1 | FOSB | -12.4518 | 0.528787994 | MIR22 | 74 | 85 | + | GGCGGAGTGGCG |
| UN0122.1 | FOSB | -12.4542 | 0.528753649 | MIR22 | 1161 | 1172 | - | CCCTGCAGCATG |
| UN0122.1 | FOSB | -12.4607 | 0.5286576 | MIR22 | 1645 | 1656 | - | ACACACCCCACC |
| UN0122.1 | FOSB | -12.4793 | 0.5283878 | MIR22 | 1645 | 1656 | + | GGTGGGGTGTGT |
| UN0122.1 | FOSB | -12.4933 | 0.528183439 | MIR22 | 1642 | 1653 | + | GCTGGTGGGGTG |
| UN0122.1 | FOSB | -12.5091 | 0.527953608 | MIR22 | 1414 | 1425 | - | GGAGGTATCGGC |
| UN0122.1 | FOSB | -12.5144 | 0.527876225 | MIR22 | 1378 | 1389 | - | GGGCAGGGGACC |
| UN0122.1 | FOSB | -12.5194 | 0.527804188 | MIR22 | 613 | 624 | + | TCTCTCCCCACC |
| UN0122.1 | FOSB | -12.5272 | 0.527689529 | MIR22 | 691 | 702 | - | GCTGGAACCAGG |
| UN0122.1 | FOSB | -12.5717 | 0.5270422 | MIR22 | 206 | 217 | + | GGCGAGGAGGTG |
| UN0122.1 | FOSB | -12.5782 | 0.526946943 | MIR22 | 1910 | 1921 | - | AACGCTGACCCA |
| UN0122.1 | FOSB | -12.6021 | 0.526599884 | MIR22 | 160 | 171 | + | GGGCCGGCCATC |
| UN0122.1 | FOSB | -12.6126 | 0.526446408 | MIR22 | 1102 | 1113 | - | GGGGAGGGCGGG |
| UN0122.1 | FOSB | -12.6237 | 0.526284572 | MIR22 | 1352 | 1363 | + | CCATACCTCCCT |
| UN0122.1 | FOSB | -12.6634 | 0.525706474 | MIR22 | 1726 | 1737 | - | GCTCTCACCCTC |
| UN0122.1 | FOSB | -12.6779 | 0.52549492 | MIR22 | 598 | 609 | - | AGCCCAGTGATG |
| UN0122.1 | FOSB | -12.6825 | 0.525428202 | MIR22 | 477 | 488 | - | TCTCTCGGCCCA |
| UN0122.1 | FOSB | -12.6838 | 0.525409203 | MIR22 | 1328 | 1339 | + | GGGGAAGCTGCC |
| UN0122.1 | FOSB | -12.6855 | 0.525384427 | MIR22 | 1209 | 1220 | + | GGGGGCGGGGCT |
| UN0122.1 | FOSB | -12.7071 | 0.525069547 | MIR22 | 674 | 685 | + | CCCCACCTCCCT |
| UN0122.1 | FOSB | -12.7142 | 0.524967332 | MIR22 | 787 | 798 | - | ACTGACCCTCTG |
| UN0122.1 | FOSB | -12.7159 | 0.524941514 | MIR22 | 35 | 46 | + | GAGGAAAGAGTC |
| UN0122.1 | FOSB | -12.7582 | 0.524326252 | MIR22 | 647 | 658 | - | GAGGCCATGGGG |
| UN0122.1 | FOSB | -12.7679 | 0.524184942 | MIR22 | 817 | 828 | - | CTTGGCTGCAGG |
| UN0122.1 | FOSB | -12.7736 | 0.524101712 | MIR22 | 787 | 798 | + | CAGAGGGTCAGT |
| UN0122.1 | FOSB | -12.7784 | 0.524031342 | MIR22 | 1271 | 1282 | + | ATTCATACCAGA |
| UN0122.1 | FOSB | -12.8084 | 0.523595164 | MIR22 | 418 | 429 | + | GAGGGCAAGAGG |
| UN0122.1 | FOSB | -12.8138 | 0.523515975 | MIR22 | 674 | 685 | - | AGGGAGGTGGGG |
| UN0122.1 | FOSB | -12.8255 | 0.523345973 | MIR22 | 828 | 839 | + | GGGCACCTGCTT |
| UN0122.1 | FOSB | -12.8255 | 0.523345529 | MIR22 | 1650 | 1661 | - | CCTGGACACACC |
| UN0122.1 | FOSB | -12.8271 | 0.523323169 | MIR22 | 1520 | 1531 | + | TCTTCCCCAATC |
| UN0122.1 | FOSB | -12.8382 | 0.523161639 | MIR22 | 1665 | 1676 | - | GGTGGGGCCCGG |
| UN0122.1 | FOSB | -12.8583 | 0.522868451 | MIR22 | 1352 | 1363 | - | AGGGAGGTATGG |
| UN0122.1 | FOSB | -12.8769 | 0.522597679 | MIR22 | 521 | 532 | - | GGCCTCACAATC |
| UN0122.1 | FOSB | -12.8774 | 0.522590137 | MIR22 | 1737 | 1748 | - | AACCCAGTCCTG |
| UN0122.1 | FOSB | -12.8786 | 0.522572458 | MIR22 | 1383 | 1394 | + | CCTGCCCGCCCC |
| UN0122.1 | FOSB | -12.8996 | 0.522266799 | MIR22 | 730 | 741 | - | CAATCTGACAGC |
| UN0122.1 | FOSB | -12.9059 | 0.522174361 | MIR22 | 1520 | 1531 | - | GATTGGGGAAGA |
| UN0122.1 | FOSB | -12.9151 | 0.522041037 | MIR22 | 828 | 839 | - | AAGCAGGTGCCC |
| UN0122.1 | FOSB | -12.9159 | 0.522030079 | MIR22 | 1871 | 1882 | - | GTAGACCTCTGA |
| UN0122.1 | FOSB | -12.9245 | 0.521903505 | MIR22 | 1642 | 1653 | - | CACCCCACCAGC |
| UN0122.1 | FOSB | -12.9329 | 0.52178193 | MIR22 | 1614 | 1625 | - | TGTGGTACCTCA |
| UN0122.1 | FOSB | -12.9415 | 0.521655953 | MIR22 | 1447 | 1458 | - | GGGGCGGACAGG |
| UN0122.1 | FOSB | -12.9467 | 0.521580542 | MIR22 | 1005 | 1016 | - | GGGTAAGAAAGA |
| UN0122.1 | FOSB | -12.9701 | 0.521239441 | MIR22 | 1871 | 1882 | + | TCAGAGGTCTAC |
| UN0122.1 | FOSB | -12.9858 | 0.521011957 | MIR22 | 1614 | 1625 | + | TGAGGTACCACA |
| UN0122.1 | FOSB | -13.0048 | 0.520734629 | MIR22 | 1921 | 1932 | - | CATGTTGCTGCA |
| UN0122.1 | FOSB | -13.0237 | 0.520459398 | MIR22 | 51 | 62 | - | CACGACTCCCGT |
| UN0122.1 | FOSB | -13.0312 | 0.520349767 | MIR22 | 1583 | 1594 | - | AGTGATCAGCCC |
| UN0122.1 | FOSB | -13.032 | 0.520338407 | MIR22 | 647 | 658 | + | CCCCATGGCCTC |
| UN0122.1 | FOSB | -13.0597 | 0.519935074 | MIR22 | 1209 | 1220 | - | AGCCCCGCCCCC |
| UN0122.1 | FOSB | -13.0817 | 0.519614666 | MIR22 | 1442 | 1453 | + | GGTCCCCTGTCC |
| UN0122.1 | FOSB | -13.0852 | 0.519563503 | MIR22 | 668 | 679 | - | GTGGGGGTGACA |
| UN0122.1 | FOSB | -13.1125 | 0.519166975 | MIR22 | 228 | 239 | + | GCCGGCGTTCAC |
| UN0122.1 | FOSB | -13.1139 | 0.519145699 | MIR22 | 1388 | 1399 | + | CCGCCCCTCACA |
| UN0122.1 | FOSB | -13.1139 | 0.519145699 | MIR22 | 1452 | 1463 | + | CCGCCCCTCACA |
| UN0122.1 | FOSB | -13.1333 | 0.518863191 | MIR22 | 1161 | 1172 | + | CATGCTGCAGGG |
| UN0122.1 | FOSB | -13.1333 | 0.518863024 | MIR22 | 1791 | 1802 | + | TTTACAGCCACC |
| UN0122.1 | FOSB | -13.1694 | 0.518337728 | MIR22 | 1634 | 1645 | + | ACCTGCGTGCTG |
| UN0122.1 | FOSB | -13.1786 | 0.518203279 | MIR22 | 1383 | 1394 | - | GGGGCGGGCAGG |
| UN0122.1 | FOSB | -13.2007 | 0.517881371 | MIR22 | 779 | 790 | + | CAGCTGGTCAGA |
| UN0122.1 | FOSB | -13.2009 | 0.517879079 | MIR22 | 1532 | 1543 | - | TCTGGGGGCCTG |
| UN0122.1 | FOSB | -13.2052 | 0.517816681 | MIR22 | 1528 | 1539 | + | AATCCAGGCCCC |
| UN0122.1 | FOSB | -13.2079 | 0.517777184 | MIR22 | 159 | 170 | - | ATGGCCGGCCCC |
| UN0122.1 | FOSB | -13.2258 | 0.517515938 | MIR22 | 580 | 591 | - | CCAGGCGCCCCA |
| UN0122.1 | FOSB | -13.2324 | 0.517420348 | MIR22 | 242 | 253 | - | CTGGCCCTGACC |
| UN0122.1 | FOSB | -13.2343 | 0.517393044 | MIR22 | 618 | 629 | + | CCCCACCTCTCG |
| UN0122.1 | FOSB | -13.2462 | 0.517219556 | MIR22 | 1170 | 1181 | - | AAAGCCGGGCCC |
| UN0122.1 | FOSB | -13.2475 | 0.517200377 | MIR22 | 1532 | 1543 | + | CAGGCCCCCAGA |
| UN0122.1 | FOSB | -13.2557 | 0.517081399 | MIR22 | 159 | 170 | + | GGGGCCGGCCAT |
| UN0122.1 | FOSB | -13.3226 | 0.51610662 | MIR22 | 1021 | 1032 | + | AGTATAGAAATT |
| UN0122.1 | FOSB | -13.3319 | 0.515971491 | MIR22 | 1853 | 1864 | - | AATGCAGGGCTG |
| UN0122.1 | FOSB | -13.3494 | 0.515715953 | MIR22 | 1442 | 1453 | - | GGACAGGGGACC |
| UN0122.1 | FOSB | -13.3923 | 0.515092497 | MIR22 | 498 | 509 | - | GGGGAGGTGCAG |
| UN0122.1 | FOSB | -13.399 | 0.51499356 | MIR22 | 1170 | 1181 | + | GGGCCCGGCTTT |
| UN0122.1 | FOSB | -13.425 | 0.5146151 | MIR22 | 1085 | 1096 | - | GCTGACACTCAG |
| UN0122.1 | FOSB | -13.4269 | 0.514588394 | MIR22 | 580 | 591 | + | TGGGGCGCCTGG |
| UN0122.1 | FOSB | -13.4323 | 0.514508969 | MIR22 | 228 | 239 | - | GTGAACGCCGGC |
| UN0122.1 | FOSB | -13.4476 | 0.514286123 | MIR22 | 1347 | 1358 | - | GGTATGGGCAGG |
| UN0122.1 | FOSB | -13.4496 | 0.514257764 | MIR22 | 730 | 741 | + | GCTGTCAGATTG |
| UN0122.1 | FOSB | -13.4573 | 0.514145411 | MIR22 | 1244 | 1255 | - | GGCCCCTCCATG |
| UN0122.1 | FOSB | -13.4661 | 0.514016809 | MIR22 | 1328 | 1339 | - | GGCAGCTTCCCC |
| UN0122.1 | FOSB | -13.4693 | 0.513970215 | MIR22 | 1085 | 1096 | + | CTGAGTGTCAGC |
| UN0122.1 | FOSB | -13.4694 | 0.513968951 | MIR22 | 214 | 225 | + | GGTGCTTTGCCT |
| UN0122.1 | FOSB | -13.4701 | 0.513958271 | MIR22 | 498 | 509 | + | CTGCACCTCCCC |
| UN0122.1 | FOSB | -13.4868 | 0.513715997 | MIR22 | 898 | 909 | - | TCCGACCTTCCT |
| UN0122.1 | FOSB | -13.5208 | 0.513220948 | MIR22 | 857 | 868 | + | GCTGCTGAGTCC |
| UN0122.1 | FOSB | -13.5308 | 0.513074903 | MIR22 | 1787 | 1798 | - | GCTGTAAACAGA |
| UN0122.1 | FOSB | -13.5326 | 0.513048432 | MIR22 | 1496 | 1507 | - | GGAGGCCTAGCG |
| UN0122.1 | FOSB | -13.5414 | 0.512920594 | MIR22 | 606 | 617 | + | GGCTGCCTCTCT |
| UN0122.1 | FOSB | -13.5502 | 0.512792506 | MIR22 | 1206 | 1217 | - | CCCGCCCCCACA |
| UN0122.1 | FOSB | -13.5554 | 0.512716345 | MIR22 | 668 | 679 | + | TGTCACCCCCAC |
| UN0122.1 | FOSB | -13.5563 | 0.512704109 | MIR22 | 1269 | 1280 | - | TGGTATGAATTC |
| UN0122.1 | FOSB | -13.5639 | 0.512593423 | MIR22 | 1388 | 1399 | - | TGTGAGGGGCGG |
| UN0122.1 | FOSB | -13.5639 | 0.512593423 | MIR22 | 1452 | 1463 | - | TGTGAGGGGCGG |
| UN0122.1 | FOSB | -13.5662 | 0.512559578 | MIR22 | 1353 | 1364 | + | CATACCTCCCTC |
| UN0122.1 | FOSB | -13.5744 | 0.512439725 | MIR22 | 1834 | 1845 | + | CGGGCCCAGATC |
| UN0122.1 | FOSB | -13.5816 | 0.512334996 | MIR22 | 898 | 909 | + | AGGAAGGTCGGA |
| UN0122.1 | FOSB | -13.5826 | 0.51232122 | MIR22 | 206 | 217 | - | CACCTCCTCGCC |
| UN0122.1 | FOSB | -13.5901 | 0.512210741 | MIR22 | 618 | 629 | - | CGAGAGGTGGGG |
| UN0122.1 | FOSB | -13.6129 | 0.511878778 | MIR22 | 1336 | 1347 | + | TGCCCAGCCACC |
| UN0122.1 | FOSB | -13.6157 | 0.511838656 | MIR22 | 1910 | 1921 | + | TGGGTCAGCGTT |
| UN0122.1 | FOSB | -13.652 | 0.51131036 | MIR22 | 1447 | 1458 | + | CCTGTCCGCCCC |
| UN0122.1 | FOSB | -13.6917 | 0.510732234 | MIR22 | 242 | 253 | + | GGTCAGGGCCAG |
| UN0122.1 | FOSB | -13.6987 | 0.510629172 | MIR22 | 268 | 279 | - | GACCCCTTCCCT |
| UN0122.1 | FOSB | -13.7171 | 0.510361774 | MIR22 | 1953 | 1964 | - | AGGGAGGGAAAA |
| UN0122.1 | FOSB | -13.7474 | 0.509920735 | MIR22 | 1953 | 1964 | + | TTTTCCCTCCCT |
| UN0122.1 | FOSB | -13.7831 | 0.5094008 | MIR22 | 954 | 965 | - | TTGGGAGGCATG |
| UN0122.1 | FOSB | -13.79 | 0.509300251 | MIR22 | 1370 | 1381 | - | GACCACAGGCTG |
| UN0122.1 | FOSB | -13.79 | 0.509300251 | MIR22 | 1434 | 1445 | - | GACCACAGGCTG |
| UN0122.1 | FOSB | -13.7906 | 0.509291571 | MIR22 | 1484 | 1495 | - | TTTGAAAGAAGG |
| UN0122.1 | FOSB | -13.8095 | 0.509016063 | MIR22 | 1297 | 1308 | + | GCAAGCGGAATG |
| UN0122.1 | FOSB | -13.8348 | 0.508648033 | MIR22 | 1496 | 1507 | + | CGCTAGGCCTCC |
| UN0122.1 | FOSB | -13.8411 | 0.508555581 | MIR22 | 488 | 499 | - | AGAGGTGACCTT |
| UN0122.1 | FOSB | -13.8562 | 0.508335749 | MIR22 | 691 | 702 | + | CCTGGTTCCAGC |
| UN0122.1 | FOSB | -13.8716 | 0.508111543 | MIR22 | 1185 | 1196 | + | TGCTGATTCATG |
| UN0122.1 | FOSB | -13.8791 | 0.508002926 | MIR22 | 1737 | 1748 | + | CAGGACTGGGTT |
| UN0122.1 | FOSB | -13.8814 | 0.507969525 | MIR22 | 1761 | 1772 | - | CTTTCCAGAACT |
| UN0122.1 | FOSB | -13.8899 | 0.50784502 | MIR22 | 1752 | 1763 | + | CTAGACTCCAGT |
| UN0122.1 | FOSB | -13.9234 | 0.507357818 | MIR22 | 1634 | 1645 | - | CAGCACGCAGGT |
| UN0122.1 | FOSB | -13.9301 | 0.507260422 | MIR22 | 291 | 302 | - | GAGGAGGGGCCG |
| UN0122.1 | FOSB | -13.9356 | 0.507180094 | MIR22 | 598 | 609 | + | CATCACTGGGCT |
| UN0122.1 | FOSB | -13.9486 | 0.506990427 | MIR22 | 1761 | 1772 | + | AGTTCTGGAAAG |
| UN0122.1 | FOSB | -13.9521 | 0.506939806 | MIR22 | 1370 | 1381 | + | CAGCCTGTGGTC |
| UN0122.1 | FOSB | -13.9521 | 0.506939806 | MIR22 | 1434 | 1445 | + | CAGCCTGTGGTC |
| UN0122.1 | FOSB | -13.9539 | 0.506912919 | MIR22 | 35 | 46 | - | GACTCTTTCCTC |
| UN0122.1 | FOSB | -13.9559 | 0.506884393 | MIR22 | 1206 | 1217 | + | TGTGGGGGCGGG |
| UN0122.1 | FOSB | -13.9624 | 0.506790441 | MIR22 | 184 | 195 | - | GGGGTCCAGACC |
| UN0122.1 | FOSB | -13.9997 | 0.50624591 | MIR22 | 1498 | 1509 | + | CTAGGCCTCCTC |
| UN0122.1 | FOSB | -14.0121 | 0.506066103 | MIR22 | 356 | 367 | - | CCCGGCACCGCC |
| UN0122.1 | FOSB | -14.0174 | 0.505988872 | MIR22 | 291 | 302 | + | CGGCCCCTCCTC |
| UN0122.1 | FOSB | -14.0336 | 0.50575325 | MIR22 | 74 | 85 | - | CGCCACTCCGCC |
| UN0122.1 | FOSB | -14.0432 | 0.505612927 | MIR22 | 801 | 812 | + | CCTGGGCCCACC |
| UN0122.1 | FOSB | -14.0435 | 0.505609205 | MIR22 | 1040 | 1051 | + | AAGACCGCCTTG |
| UN0122.1 | FOSB | -14.0448 | 0.505589581 | MIR22 | 1791 | 1802 | - | GGTGGCTGTAAA |
| UN0122.1 | FOSB | -14.0462 | 0.50556911 | MIR22 | 606 | 617 | - | AGAGAGGCAGCC |
| UN0122.1 | FOSB | -14.0498 | 0.505516697 | MIR22 | 881 | 892 | + | AGAGCTATCTCA |
| UN0122.1 | FOSB | -14.0631 | 0.505323989 | MIR22 | 1185 | 1196 | - | CATGAATCAGCA |
| UN0122.1 | FOSB | -14.1013 | 0.504766417 | MIR22 | 42 | 53 | + | GAGTCTGGGACG |
| UN0122.1 | FOSB | -14.1032 | 0.504739946 | MIR22 | 1528 | 1539 | - | GGGGCCTGGATT |
| UN0122.1 | FOSB | -14.1049 | 0.504714309 | MIR22 | 318 | 329 | - | TTACATAACATC |
| UN0122.1 | FOSB | -14.1058 | 0.504702199 | MIR22 | 165 | 176 | - | GGCTAGATGGCC |
| UN0122.1 | FOSB | -14.1161 | 0.504551751 | MIR22 | 980 | 991 | + | TTGAACCGCACC |
| UN0122.1 | FOSB | -14.1262 | 0.504403734 | MIR22 | 860 | 871 | - | AGAGGACTCAGC |
| UN0122.1 | FOSB | -14.1346 | 0.504281659 | MIR22 | 839 | 850 | - | GAGTCTGTGCCA |
| UN0122.1 | FOSB | -14.1369 | 0.504248981 | MIR22 | 1842 | 1853 | - | GCAGCTCTGATC |
| UN0122.1 | FOSB | -14.151 | 0.504043634 | MIR22 | 182 | 193 | - | GGTCCAGACCCT |
| UN0122.1 | FOSB | -14.1569 | 0.503956696 | MIR22 | 1498 | 1509 | - | GAGGAGGCCTAG |
| UN0122.1 | FOSB | -14.1776 | 0.503656522 | MIR22 | 1110 | 1121 | + | CCCCAGCTCACA |
| UN0122.1 | FOSB | -14.1829 | 0.503578014 | MIR22 | 1583 | 1594 | + | GGGCTGATCACT |
| UN0122.1 | FOSB | -14.1879 | 0.503505602 | MIR22 | 758 | 769 | - | CCCTCTGGCACT |
| UN0122.1 | FOSB | -14.2064 | 0.503236941 | MIR22 | 1698 | 1709 | - | CCTGGGGGCCCA |
| UN0122.1 | FOSB | -14.2384 | 0.502771071 | MIR22 | 234 | 245 | - | GACCCAGTGAAC |
| UN0122.1 | FOSB | -14.2392 | 0.502759516 | MIR22 | 1040 | 1051 | - | CAAGGCGGTCTT |
| UN0122.1 | FOSB | -14.2486 | 0.502622248 | MIR22 | 1131 | 1142 | + | CCTGCCACGCCC |
| UN0122.1 | FOSB | -14.2495 | 0.502609304 | MIR22 | 1336 | 1347 | - | GGTGGCTGGGCA |
| UN0122.1 | FOSB | -14.2538 | 0.502546489 | MIR22 | 801 | 812 | - | GGTGGGCCCAGG |
| UN0122.1 | FOSB | -14.2685 | 0.502332407 | MIR22 | 881 | 892 | - | TGAGATAGCTCT |
| UN0122.1 | FOSB | -14.2762 | 0.502219693 | MIR22 | 758 | 769 | + | AGTGCCAGAGGG |
| UN0122.1 | FOSB | -14.3105 | 0.501720589 | MIR22 | 418 | 429 | - | CCTCTTGCCCTC |
| UN0122.1 | FOSB | -14.313 | 0.501684355 | MIR22 | 1297 | 1308 | - | CATTCCGCTTGC |
| UN0122.1 | FOSB | -14.3159 | 0.501642275 | MIR22 | 1375 | 1386 | - | CAGGGGACCACA |
| UN0122.1 | FOSB | -14.3159 | 0.501642275 | MIR22 | 1439 | 1450 | - | CAGGGGACCACA |
| UN0122.1 | FOSB | -14.3288 | 0.501454233 | MIR22 | 1269 | 1280 | + | GAATTCATACCA |
| UN0122.1 | FOSB | -14.329 | 0.50145158 | MIR22 | 22 | 33 | + | TGTCAGGAGCTA |
| UN0122.1 | FOSB | -14.3382 | 0.501317506 | MIR22 | 165 | 176 | + | GGCCATCTAGCC |
| UN0122.1 | FOSB | -14.3396 | 0.501297146 | MIR22 | 1501 | 1512 | - | AGGGAGGAGGCC |
| UN0122.1 | FOSB | -14.3436 | 0.501239137 | MIR22 | 946 | 957 | - | CATGAACTTTGT |
| UN0122.1 | FOSB | -14.3554 | 0.501066301 | MIR22 | 980 | 991 | - | GGTGCGGTTCAA |
| UN0122.1 | FOSB | -14.3622 | 0.500967114 | MIR22 | 42 | 53 | - | CGTCCCAGACTC |
| UN0122.1 | FOSB | -14.3635 | 0.500949351 | MIR22 | 493 | 504 | + | CACCTCTGCACC |
| UN0122.1 | FOSB | -14.3767 | 0.500756157 | MIR22 | 922 | 933 | - | CCCTGGGTGACC |
| UN0122.1 | FOSB | -14.3816 | 0.500685314 | MIR22 | 1110 | 1121 | - | TGTGAGCTGGGG |
| UN0122.1 | FOSB | -14.3948 | 0.500492439 | MIR22 | 356 | 367 | + | GGCGGTGCCGGG |
| UN0122.1 | FOSB | -14.4112 | 0.500254358 | MIR22 | 503 | 514 | - | GCTGGGGGGAGG |
| UN0122.1 | FOSB | -14.4131 | 0.500226805 | MIR22 | 737 | 748 | - | CCTAGCCCAATC |
| UN0122.1 | FOSB | -14.4134 | 0.500221666 | MIR22 | 634 | 645 | - | GGGGCCAAGAGT |
| UN0122.1 | FOSB | -14.4161 | 0.500182919 | MIR22 | 1239 | 1250 | + | TCAGACATGGAG |
| UN0122.1 | FOSB | -14.4179 | 0.500156838 | MIR22 | 839 | 850 | + | TGGCACAGACTC |
| UN0122.1 | FOSB | -14.4211 | 0.500109591 | MIR22 | 106 | 117 | - | CCCTCCGGCCCC |
| UN0122.1 | FOSB | -14.4264 | 0.500032735 | MIR22 | 940 | 951 | + | AGATACACAAAG |
